# Oral and injectable opioid agonist treatments for people who use street opioids: a systematic literature review and network meta-analysis

**DOI:** 10.1186/s12889-025-24365-w

**Published:** 2025-08-30

**Authors:** Nick Bansback, Alexander C.T. Tam, Heather Palis, Steve Kanters, Evan Popoff, Martin T. Schechter, Aslam H. Anis, David C. Marsh, Eugenia Oviedo-Joekes

**Affiliations:** 1 School of Population and Public Health, Faculty of Medicine, University of British Columbia, Vancouver, BC Canada; 2 Centre for Advancing Health Outcomes, Providence Research, St. Paul’s Hospital, Vancouver, BC Canada; 3 British Columbia Centre for Disease Control, Vancouver, BC Canada; 4 RainCity Analytics, Vancouver, BC Canada; 5 NOSM University, Sudbury, ON Canada; 6 ICES North, Sudbury, ON Canada; 7 Health Sciences North Research Institute, Sudbury, ON Canada

**Keywords:** Opioid-Related Disorders[MeSH], Opioid agonist treatment, Methadone[MeSH], Buprenorphine[MeSH], Treatment retention

## Abstract

**Objective:**

To synthesize and determine the relative effectiveness of diverse opioid agonist treatment (OAT) medications, including injectables, for opioid use disorder (OUD).

**Methods:**

We searched EMBASE, PubMed, and CENTRAL for Randomised Controlled Trials (RCTs) (CRD42018109469) and previously published systematic reviews of head-to-head trials of OAT medications. The primary outcome was treatment retention, and secondary outcomes included days of opioid use, days of cocaine use, and proportion of participants involved in criminalized activities. We calculated odds ratios (ORs) and mean differences (MDs) and corresponding 95% credible intervals (CrI) using Bayesian network meta-analyses (NMAs) to indirectly compare treatments at varying lengths of follow-up (3 to 12 months). Sensitivity analyses examined influence of follow-up duration and other trial factors.

**Results:**

Twenty-four RCTs were included. Diacetylmorphine plus oral methadone and injectable hydromorphone plus oral methadone had similar retention compared to one another (OR: 1.05; 95%CrI: 0.27, 4.10). Diacetylmorphine plus oral methadone had similar or statistically favourable retention versus low, medium, and high doses of conventional OATs: buprenorphine (OR: 13.55; 95%CrI: 4.51, 42.52; OR: 5.07; 95%CrI: 2.03, 12.47; OR: 2.21; 95%CrI: 0.18, 21.54) and methadone (OR: 5.88; 95%CrI: 2.34, 16.33; OR: 3.66; 95%CrI: 1.57, 8.82; OR: 3.67; 95%CrI: 1.83, 8.35). Similarly, injectable hydromorphone plus oral methadone also showed favourable or similar retention relative to conventional OATs. Limiting analyses to trials that included only OAT-experienced patients, that offered no extra participation incentive, and/or with 6 months (± 0.5) of follow-up generally did not change the direction of the findings. Injectable hydromorphone plus oral methadone was also statistically favoured in terms of reduced days of opioid use relative to methadone, but mean differences in days of cocaine use were similar. Diacetylmorphine plus oral methadone was associated with a smaller proportion of participation in criminalized activities relative to methadone alone.

**Conclusion:**

Both diacetylmorphine and injectable hydromorphone supplemented with methadone showed favourable retention compared to methadone and buprenorphine, depending on the strength of the OAT being co-prescribed or being compared to. These results provide further support for alternatives to conventional OATs such as diacetylmorphine or injectable hydromorphone for treatment retention.

**Supplementary Information:**

The online version contains supplementary material available at 10.1186/s12889-025-24365-w.

## Introduction

Opioid use disorder (OUD) is characterized by patterns of continued opioid use and intervening periods of treatment, abstinence, and relapse [1]. It continues to be a major public health concern, particularly in North America, and is correlated with harms to the individual, their family, and their communities [2–4]. The ongoing drug toxicity (i.e., overdose) crisis has further exposed and exacerbated many fundamental failures and limitations of current approaches [5], among them the urgent need to improve access to and quality of care for people with OUD [6]. Due in part to the chronic and relapsing nature of OUD, pharmacotherapies, often integrated with psychosocial services, are an essential part of a long-term comprehensive treatment strategy [7].

Opioid agonist treatment (OAT) with long-acting oral opioids, the most widely used pharmacological therapy for OUD, aims at preventing withdrawal, alleviating cravings, and reducing use of “street” (i.e. illicitly manufactured or otherwise criminalized, and often of unpredictable doses) drugs. Oral OAT also aims at reducing participation in other criminalized behaviours, improving health and quality of life, and reducing the overall harms associated with use of street drugs (and imposed upon people who use them) [8–10]. Orally administered methadone is the most widely studied and prescribed OAT. At doses of 60 mg or more, systematic reviews have identified oral methadone to be an effective form of treatment for OUD compared to placebo or lower doses, in terms of treatment retention and reduction in the use of street opioids [11, 12]; particularly when doses are flexible as opposed to fixed [13]. Buprenorphine, a partial opioid agonist with demonstrated efficacy in the treatment of OUD [13], is the second most prescribed medication worldwide and is preferred by some patients [14]. Sublingual buprenorphine combined with naloxone, has gained preference by the health care system in the last decade due to it safety profile, ability to rapidly titrate to an effective dose and flexible dosing [15]. Depot buprenorphine, a long-acting injectable formulation, offer similar pharmacological efficacy with less frequent dosing [16]. This route may enhance adherence and autonomy among some patients, and is increasingly being adopted in clinical practice [17, 18].

Despite their effectiveness, well-established dosing, and inclusion in clinical guidelines, oral methadone and sublingual buprenorphine remain underutilized. Fewer than 30% initiate treatment and less than half are retained beyond six months [19–21], limiting their benefit for many patients. The chronic nature of OUD, characterized by periods of remission and recurrence, further complicates sustained treatment engagement, especially when compounded by programmatic rigidity and systemic barriers such as stigma or unstable housing [21], including lack of pharmaceutical alternatives to match individual needs or preferences.Those not retained in treatment remain at very high risk of lethal and non-lethal harm [22–25]. This risk has been compounded by the advent of increasingly potent opioids such as fentanyl, carfentanil, and other synthetic opioids and their analogues in the illicit market [26] – the source that most people who use opioids rely upon. The scale of the crisis has contributed to an increasing focus on the study of alternative treatments for OUD, such as slow-release oral morphine, injectable diacetylmorphine (the active ingredient in heroin), and injectable hydromorphone.

A challenge with comparing the evidence for treatments with medications that have different active pharmaceutical ingredients (e.g., buprenorphine, hydromorphone) is that oral methadone is used as the comparator in most published randomized controlled trials (RCTs). Therefore, there are few head-to-head comparisons of those other medications relative to one another – despite evidence of superior treatment outcomes [27]. For example, the only medication other than methadone that has been compared with injectable diacetylmorphine in an RCT, is injectable hydromorphone [28], and it has itself not been directly compared with methadone (with the exception of a small group of patients in the North American Opiate Medication Initiative [NAOMI] study) [29]. As the superiority efficacy profile of several medications used in OAT remain poorly understood, a recent network meta-analysis (NMA) of OAT investigated the comparative retention of medications for OUD [30]. NMA is an extension of traditional meta-analysis, whereby multiple pairwise comparisons are conducted, involving three or more interventions [31]. As expected, all treatments had higher retention than non-pharmacological controls. Methadone was superior to buprenorphine and buprenorphine to naltrexone. However, importantly this NMA did not consider the dose (e.g. personalized vs. fixed dose) as part of effectiveness, and did not include injectable OAT in the comparisons, even when there are several RCTs comparing injectable diacetylmorphine and oral methadone [30].

Evidence from NMA can support clinical practice guidelines [32] by providing comparative benefit and risk profiles across all potential treatment options by synthesizing the evidence base and contribute to the identification of priority treatment comparisons in clinical trials [33]. As such, the objective of this study was to determine the relative effectiveness of OATs, at different doses, among people with OUD who use injectable street opioids, and were either not attracted or sufficiently benefiting from available OAT. We use an NMA to make indirect comparisons between interventions, including those for which head-to-head comparisons have not been conducted.

## Materials and methods

### Search methods, trial identification and data extraction

Systematic searches were conducted in EMBASE, PubMed, and the Cochrane Central Register of Controlled Trials (CENTRAL) on 25 October 2024 to identify randomized controlled trials of OAT medications for people with OUD (PROSPERO # CRD42018109469). Relevant publications were also identified through their inclusion in previous systematic reviews [13, 27, 34, 35]. The search strategy was adapted from these previous systematic reviews to include all relevant treatments. Specifically, these were oral methadone, sublingual buprenorphine, buprenorphine/naloxone, depot injection naltrexone, slow-release oral morphine, injectable diacetylmorphine [including co-prescription with oral methadone], and injectable hydromorphone). However, the search strategies can only target the medication by their active ingredient name, whereas trials treatments could have ranged in terms of dosing and frequency, as well by routes of administration. As such, for example, identifying “buprenorphine” could later lead it to being subclassified as sublingual or depot injectable buprenorphine. This strategy was assumed to help ensure we had conducted a comprehensive search. The trials were identified from the previously conducted systematic reviews covering up to 2013 and from an updated literature search that we conducted. The updated literature search to complement previous systematic reviews was limited to English-language publications published from January 2013 to October 2024.

Inclusion was determined using PICOS (population, intervention, comparator, outcomes and study design) criteria (Additional file 1 - S1 Text). Publications had to report on a randomized control trial (RCT) using at least one OAT medication for a minimum of 3 months in a population of non-incarcerated, non-pregnant, people with OUD who were seeking treatment where a majority were people that used heroin or other injectable street opioids (e.g. fentanyl) at the time of recruitment. Trials also had to have included populations of people with previous OAT experience. Mixed populations of people with previous OAT experience and no previous OAT experience were also eligible (i.e., trials that did not have previous OAT experience as an inclusion criteria). The interventions were listed above and outcomes are specified below. Trials were excluded if they used tapering, detoxification, or different routes of administration of the same medication only, or if the trial study population included only people with no previous OAT experience. Although this focus excluded studies with more diverse OUD populations, we prioritized trials involving individuals using injectable street opioids to reflect those for whom standard treatments are often insufficient. This group commonly faces structural barriers such as housing instability [36], criminalization [37], and comorbidities [38], and centering their experiences helps identify limitations in existing treatment models and inform improvements across the broader OAT system.

Four reviewers (NB, JC, SB, and AT) were involved in the study search and selection process. All records were screened and relevant records were reviewed in full by two of the reviewers. Therefore, despite having four reviewers, all selection steps were conducted in dual and independently in accordance with PRISMA guidelines [39]. Authors were contacted if information about the population was not reported. Inconsistencies in screening decisions between the investigators were resolved by discussion and review of the material with a fifth investigator (DG or HP). We followed PRISMA checklist for reporting (Additional File 1 - S1 Text).

### Risk of bias assessment

Risk of bias was assessed using the updated Cochrane Collaboration’s tool for assessing risk bias in randomized trials [40]. Five bias domains were considered: (1) bias arising from the randomization process; (2) bias due to deviations from intended interventions; (3) bias due to missing outcome data; (4) bias in measurement of the outcome; (5) bias in selection of the reported result. Domains for each trial were assessed as having high risk of bias, some concerns, or low risk of bias [40]. Six trials were previously assessed. The assessment for the trials were conducted by two investigators (two among NB, JC, and AT) and any discrepancies were resolved by consensus with input from a fourth reviewer (WZ).

### Outcome measures

The outcomes of interest included: retention (proportion of individuals retained in the assigned treatment), street opioid use (number of days in the prior month), cocaine use (days using cocaine in the prior month), and involvement with criminalized activity (proportion of subjects engaging in criminalized activities, excluding street opioid use and cocaine use, in the prior month). Where available, data were extracted for the 6th month of follow-up; otherwise, data for other months of follow-up were extracted.

### Evidence synthesis

We conducted network meta-analyses within a Bayesian framework [41]. Logit models were used for the retention and proportion of illegal activities outcomes, while linear models were used for the other outcomes (i.e. cocaine use, street opioid use). Both random and fixed effects models were conducted for all the outcomes where possible. Meta-regressions (binary 0/1) for treatment experience were also conducted as sensitivity analyses where data allowed. As is common for NMA and evidence synthesis more generally, no imputation of outcomes was performed. Studies not reporting a particular outcome were not included in the analyses for those outcomes.

We used the deviance information criterion (DIC) for the selection between fixed and random effect models. Heterogeneity between trials was assessed using the posterior median between-trial standard deviation with its 95% credible interval (CrI). Consistency between the direct comparison and indirect comparison was assessed using the node-splitting method [42].

Relative treatment effects were reported as posterior medians of the odds-ratios (ORs) and 95% CrIs for retention and criminalized activity, and as posterior medians of the mean of differences (MDs) for street opioids and cocaine use. Differences between treatments were judged to be statistically significant when credible intervals of odds ratios did not cross 1 or CrIs of mean differences did not cross 0. Otherwise, differences were interpreted as nonsignificant.

Our primary analysis was based on data from RCTs of OAT medications for a population of people with OUD and mixed OAT experience who use heroin or similar injectable street opioids and reported on retention, use of street opioids, use of cocaine, and/or involvement in criminalized activities. Included in the primarily analysis were data from trials where previous receipt of OAT was an inclusion criterion as well as data from trials where this was not an inclusion criterion. Recognizing that there might be heterogeneity with regards to trial-level factors, such as some trials offering extra benefit to individuals in the usual care (methadone) arm at the end of the trial, or with regards to prior treatment experience, we conducted the following sensitivity analyses of subgroups to control for heterogeneity in trial design:

1. Excluding all trials in which previous treatment experience was not a criterion (i.e., there is a ‘mix’ of prior OAT experience among participants).
2. Conducting a meta-regression based on prior treatment experience.
3. Excluding all trials conducted at time points other than 6 (± 0.5) months.
4. Excluding all trials that offered extra benefits for completing the trial study (e.g., control group being offered to switch to treatment with injectable diacetylmorphine after completion).

## Results

### Systematic review and trial characteristics

**Fig. 1:**
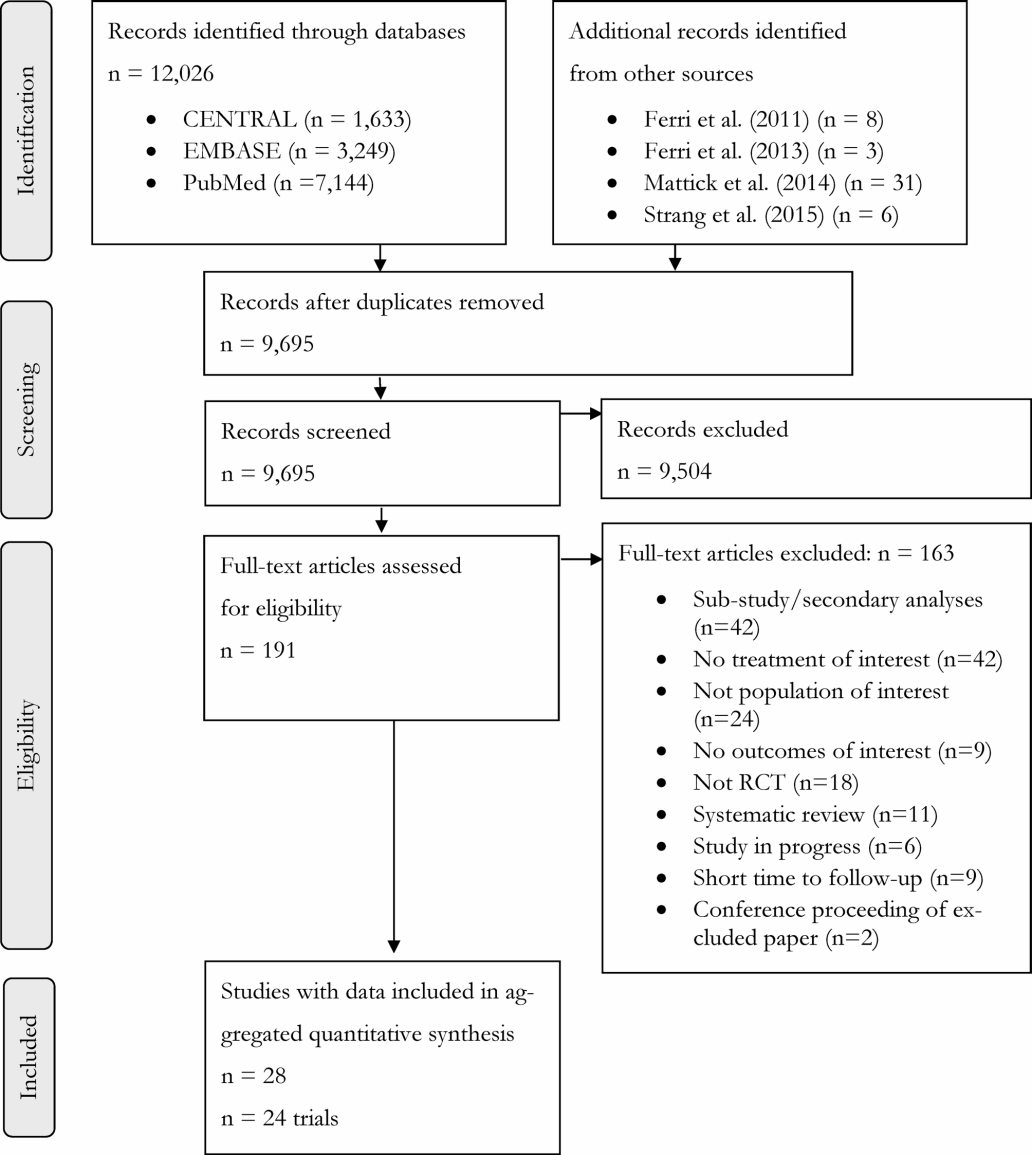
PRISMA flow diagram of studies for network meta-analysis.

After removing duplicates, a total of 9,695 articles were identified through the systematic review, as shown in Fig. 1. Following both screening stages, 28 articles reporting on 24 trials were eligible for inclusion in our analysis [28, 29, 43–68]. Trial characteristics are noted in Table 1. Methadone and buprenorphine were the most studied OAT medications, whereas injectable hydromorphone and depot injectable naltrexone were only included in 2 trials each. In terms of routes of administration, all included trials of buprenorphine were sublingual and so, buprenorphine was described as sublingual buprenorphine in subsequent sections. Among methadone studies, there was 1 trial in which route of administration information was not available [69] and 2 trials of both injectable and oral [47, 57]. Thus, while oral methadone was the predominant route studied, we did not append route of administration information to methadone when describing this OAT in subsequent sections. Similarly, diacetylmorphine was also predominantly studied as an injectable, but 1 trial offered participants the choice between inhalable and injectable [68]. As a result, we chose to refer to diacetylmorphine as diacetylmorphine without further reference to route of administration.

**Table 1:** Characteristics of included trials

Study ID	Treatment	Number randomized	Follow-up duration (weeks)	Start Date	Extra treatment benefit offered for completion	Setting
Perneger et al. 1998 [69]	Heroin	27	26	1995	Control group: priority heroin maintenance program access	Switzerland
	Methadone/detoxification/residential	24				
van den Brink et al. 2003 [57]	Heroin + Methadone (M)(V)	76	52	1998	Control group: methadone + heroin for 6 months	The Netherlands
	Methadone (M)(V)	98				
March et al. 2006 [44]	Heroin + Methadone (M)(V)	31	39	2003	Compassionate use for heroin	Spain
	Methadone (H)(V)	31				
Haasen et al. 2007 [45, 46]	Heroin + Methadone (L)(V)	515	52	2002	–	Germany
	Methadone (M) then (H)(V)	500				
Oviedo-Joekes et al. 2009 [29]	Heroin	115	52	2005	–	Canada
	Methadone (H)(V)	111				
	Hydromorphone	25				
Strang et al. 2010 [47, 66]	Heroin + Methadone (M)(V)	43	26	2005	–	UK
	Methadone (H)(V)	42				
	Methadone (M)(V)	42				
Demaret et al. 2015 [48, 68]	Heroin + Methadone (L)(V)	36	52	2011	–	Belgium
	Methadone (M)(V)	38				
Oviedo-Joekes et al. 2016 [28]	Heroin + Methadone (L)(V)	102	26	2011	–	Canada
	Hydromorphone + Methadone (L)(V)	100				
Potteret al. 2013 [43]	Methadone (H)	529	24	2006	–	USA
	Buprenorphine (M)(V) + Naloxone	740				
Mattick et al. 2003 [49]	Methadone (M)(V)	205	13	1998	–	Australia
	Buprenorphine (M)(V)	200				
Soyka et al. 2008 [50]	Methadone (M)(V)	76	26	–	–	Germany
	Buprenorphine (M)(V)	64				
Lintzeris et al. 2004a [51]	Methadone (M)(V)	30	52	–	–	Australia
	Buprenorphine (M)(V)	27				
Lintzeris et al. 2004b [51]	Methadone (M)(V)	36	52	–	–	Australia
	Buprenorphine (M)(V)	46				
Ling et al. 1996 [52]	Methadone (H)(F)	75	52	1990	–	USA
	Methadone (L)(F)	75				
	Buprenorphine (M)(F)	75				
Neri et al. 2005 [59]	Methadone (H)(F)	31	52	2002	–	Italy
	Buprenorphine (H)(V)	31				
Pani et al. 2000 [53]	Methadone (M)(F)	34	26	–	–	Italy
	Buprenorphine (M)(F)	38				
Fischer et al. 1999 [55]	Buprenorphine (L)(V)	29	24	–	–	Austria
	Methadone (M)(V)	31				
Schottenfeld et al. 2005a [56]	Methadone (H)(V) + contingency (voucher)	40	24	1995	Both groups: continue or begin methadone after study	USA
	Buprenorphine (M)(V) + contingency (voucher)	39				
Schottenfeld et al. 2005b [56]	Methadone (H)(V) + performance counseling	40	24	1995	Both groups: continue or begin methadone after study	USA
	Buprenorphine (M)(V) + performance counseling	43				
Jutras-Aswad et al. 2022 [62]	Methadone (H)(V)	134	24	2017	–	Canada
	Buprenorphine (M)(V) + Naloxone	138				
Oliveto et al. 1999_des [64]	Buprenorphine (M)(F) + desipramine HCL	45	13	–	All groups: referral to program of choice	USA
	Methadone (M)(F) + desipramine HCL	45				
Oliveto et al. 1999_pla [64]	Buprenorphine (M)(F) + placebo	45				
	Methadone (M)(F) + placebo	45				
Opheim et al. 2021 [65, 67]	Buprenorphine (M)(V) + Naloxone	72	12	2012	–	Norway
	Naltrexone	71				
Lee et al. 2018 [58]	Buprenorphine (M)(V) + Naloxone	287	24	2014	–	USA
	Naltrexone	283				
Johnson et al. 1992 [63]	Methadone (L)(F)	55	17	1988	–	USA
	Methadone (M)(F)	54				
	Buprenorphine (M)(F)	53				
Schottenfeld et al. 1997a [54]	Methadone (L)(F)	30	24	1991	–	USA
	Buprenorphine (L)(F)	29				
Schottenfeld et al. 1997b [54]	Methadone (M)(F)	28	24	1991	–	USA
	Buprenorphine (M)(F)	29				
Kosten et al. 1993 [61]	Methadone (L)(F)	34	24	–	Both groups: continue on program or switch to program of choice	USA
	Methadone (M)(F)	35				
	Buprenorphine (L)(F)	56				

Abbreviations L, M, and H refer to low-, medium-, and high-dose versions of the medications. For methadone: high-dose = > 80 mg/day, medium-dose = 40 to 80 mg/day, and low-dose = < 40 mg/day. For sublingual buprenorphine: high-dose > 24 mg/day, medium-dose 8-24 mg/day, and low-dose < 8 mg/day. Abbreviations V and F refer to variable- and fixed-dose versions of the medications

Earlier trials (1990’s to early 2000’s) tended to implement fixed dosages for trial medications (i.e., all patients are administered the same dose). By contrast, newer trials tended to use individually titrated or personalized dosages of trial medications. The dosage (in the case of newer trials, the mean dosage) also varied across studies. Trial follow-up ranged from 3 months to 12 months. The quality assessment of the 24 trials rated 7 trials at low risk of bias; 10 were rated with some concerns; and 7 were rated at high risk of bias (Additional file 1 - S1 Table). Trials that were rated at higher risk of bias were generally noted to have deviations from intended interventions or a lack of reporting on a priori statistical analysis plans. Funnel plots were used to explore publication bias according to the four outcomes (Additional file 1 - S1 Fig). While asymmetry was not visually present for the retention outcome (S1 Fig panel B-D), the small number of studies reporting the other three outcomes precluded judgements about publication bias.

### Treatment networks

Given methadone (predominantly oral) and sublingual buprenorphine regimen definitions varied across studies, we considered two primary treatment networks: one where nodes were defined at the medication level only (e.g., methadone, sublingual buprenorphine); and, a second where nodes of the same medication were separated by their strength (e.g., high-dose methadone, medium-dose methadone). Dose strength information was only categorized for methadone and sublingual buprenorphine. For methadone, the categories were defined as high > 80 mg/day, medium 40 to 80 mg/day, and low < 40 mg/day. For sublingual buprenorphine, the categories were defined as high > 24 mg/day, medium 8-24 mg/day, and low < 8 mg/day. The categories were selected after reviewing clinical guidance from different jurisdictions suggesting 8-24 mg as a typical range at stabilization for buprenorphine and 60-120 mg for methadone [15, 70, 71], as well as reviewing other reviews using different indicating > 80 mg or > 85 mg as a “high” dose and < 40 mg as a “low” dose for methadone [12, 13, 71, 72].

**Fig. 2:**
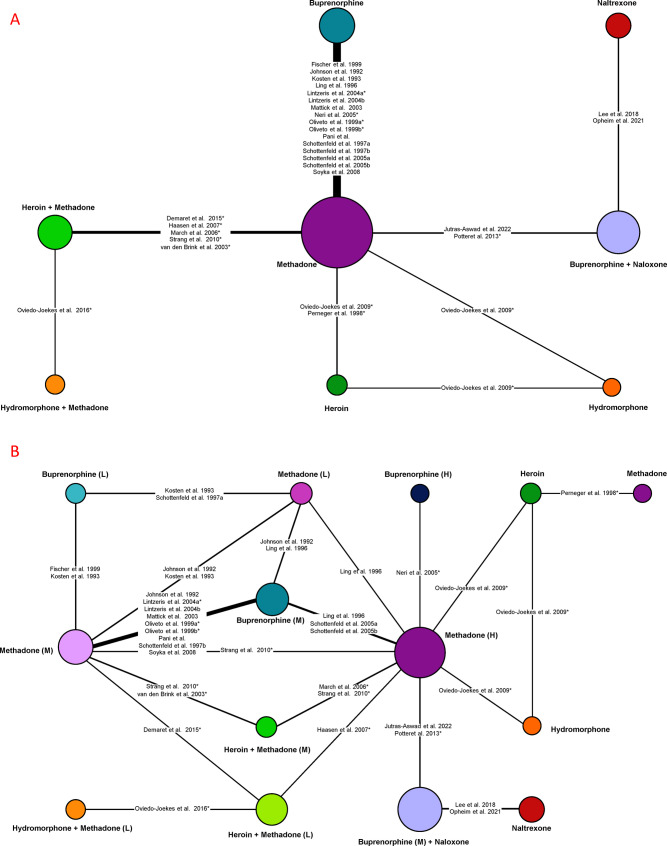
Treatment networks. Legend: Abbreviations _L, _M, and _H refer to low-, medium-, and high-dose versions of the medications

Treatment networks for analyses of the retention outcome are presented in Fig. 2. No trials were removed due to being disconnected from networks.

### Model selection

Previous treatment experience was found to be a prognostic factor in a pairwise meta-analysis, and thus meta-regression models were conducted where data were available. Based on the DIC, a random-effect meta-regression model was selected for the retention outcome. Fixed-effect models without meta-regression were selected for the other three outcomes given sparse data. Of note, all networks met the NMA conditions including the consistency assumption. Across the main networks which used random-effects models, between-study heterogeneity (τ) (95% CrI) ranged from 0.35 (0.07, 0.66) to 0.54 (0.31, 0.87). These values suggest low to moderate heterogeneity, consistent with expectations for continuous outcomes. A single heterogeneity parameter was assumed across comparisons within each network.

### Retention

**Fig. 3:**
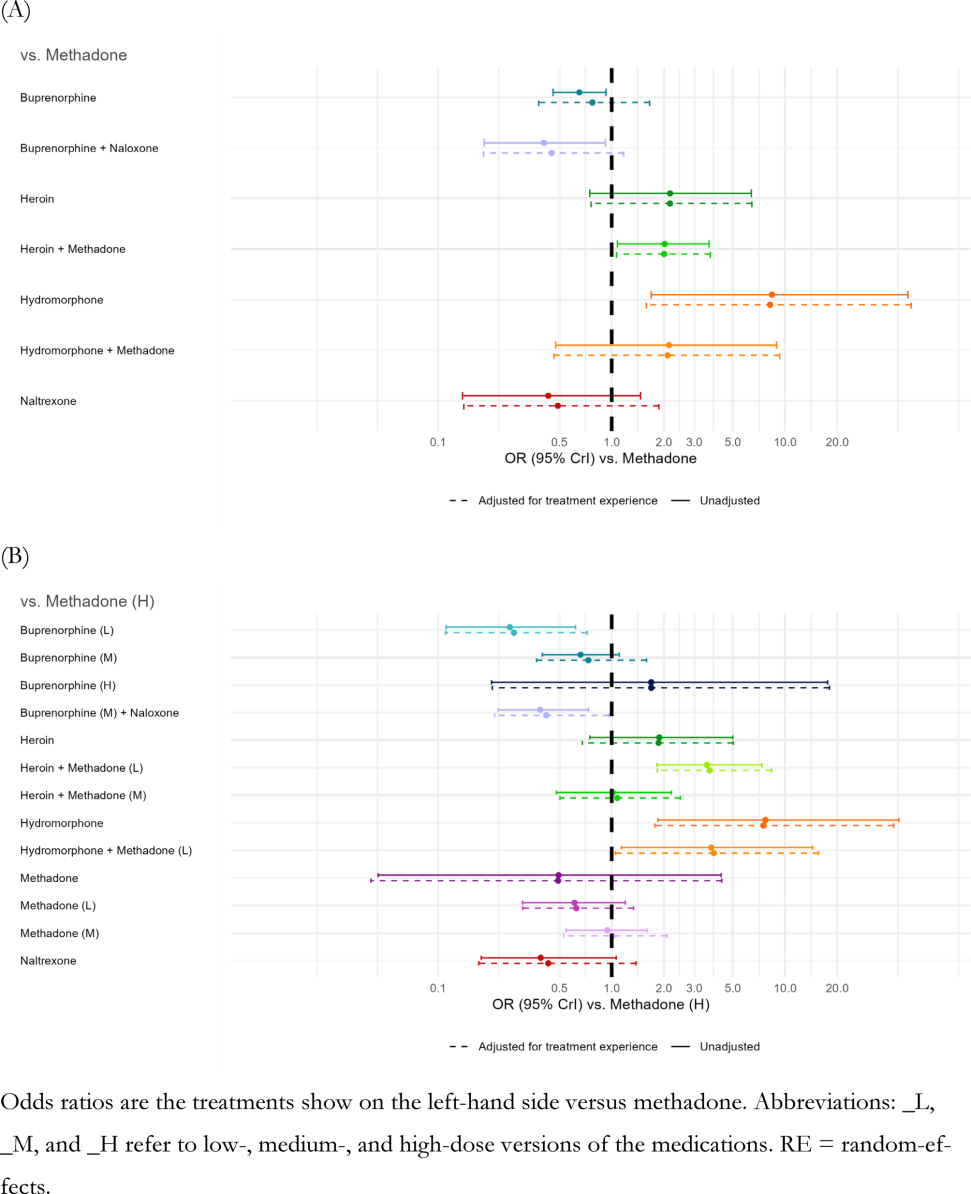
Comparative effects (odds ratios and 95% credible intervals) of treatment retention at 3 to 12 months of follow-up

Retention was often defined as either attending the final trial follow-up visit or by completing the planned treatments (Additional file 1 - S2 Table). We narrowed our focus to per protocol results of the trials as our outcome of interest was retention in the *assigned* treatment. In the treatment network where OATs were defined at the medication level (Fig. 3A), neither sublingual buprenorphine nor methadone were statistically favoured over the other; however, point estimates favoured methadone for treatment retention (OR: 1.29; 95% CrI: 0.60, 2.63) (Additional file 1 - S3 Table). Diacetylmorphine supplemented with optional oral methadone was statistically favoured over methadone alone (OR: 2.01; 95% CrI: 1.07, 3.71) and buprenorphine/naloxone (OR: 4.45; 95% CrI: 1.39, 13.19). While non-statistically significant, the direction of ORs were greater than 1 for injectable hydromorphone supplemented with optional oral methadone relative to conventional OATs (methadone (OR: 2.10; 95% CrI: 0.46, 9.31); sublingual buprenorphine (OR: 2.71; 95% CrI: 0.50, 14.01); buprenorphine/naloxone (OR: 4.69; 95% CrI: 0.77, 26.39)); whereas, ORs relative to diacetylmorphine and diacetylmorphine supplemented with optional oral methadone were close to 1.

When the network was expanded to consider medication strength, some patterns in point estimates emerged (Fig. 3B, Additional file 1 - S4 Table). Higher dosing within either drug showed better retention to assigned treatment relative to lower dose categories of the same drug, although most associations were also non-statistically significant: high-dose sublingual buprenorphine was favoured over medium- and low-dose sublingual buprenorphine (OR: 2.31; 95% CrI: 0.24, 26.74; OR: 6.20; 95% CrI: 0.58, 76.87, respectively); medium-dose sublingual buprenorphine was favoured over low-dose sublingual buprenorphine (OR: 2.68; 95% CrI: 1.17, 6.33); high- and medium-dose methadone was favoured over low-dose methadone (OR: 1.60; 95% CrI: 0.75, 3.25; OR: 1.62; 95% CrI: 0.86, 3.15, respectively). The exception was the comparison of high-dose methadone and medium-dose methadone, which showed similar retention. The high-dose categories for either drug showed better retention relative to low-dose categories of the other drug: high-dose methadone versus low-dose sublingual buprenorphine (OR: 3.66; 95% CrI: 1.39, 9.06); high-dose sublingual buprenorphine versus low-dose methadone (OR: 2.69; 95% CrI: 0.27, 31.59).

Data were only available for one strength of injectable hydromorphone supplemented with low-dose oral methadone and direction of point estimates against different strengths of conventional OAT were consistent with the directions observed in the network defined at the medication level. However, several associations became statistically significant: injectable hydromorphone supplemented with low-dose oral methadone was statistically favourable over low- and medium-dose sublingual buprenorphine (OR: 14.21; 95% CrI: 2.98, 69.52; OR: 5.30; 95% CrI: 1.26, 22.28, respectively); medium-dose buprenorphine/naloxone (OR: 9.23; 95% CrI: 2.04, 41.05); low- and high-dose methadone (OR: 6.25; 95% CrI: 1.49, 27.50; OR: 3.88; 95% CrI: 1.05, 15.59, respectively); and, depot injectable naltrexone (OR: 8.98, 95% CrI: 1.62, 43.95).

Diacetylmorphine supplemented with low-dose oral methadone had statistically significantly better retention to assigned treatment compared to low, medium, and high doses of methadone (OR: 5.88; 95% CrI: 2.34, 16.33; OR: 3.66; 95% CrI: 1.57, 8.82; OR: 3.67; 95% CrI: 1.83, 8.35, respectively); low and medium doses of sublingual buprenorphine (OR: 13.55; 95% CrI: 4.51, 42.52; OR: 5.07; 95% CrI: 2.03, 12.47, respectively); buprenorphine/naloxone (OR: 8.80; 95% CrI: 3.11, 23.96); and, depot injectable naltrexone (OR: 8.54; 95% CrI: 2.30, 27.93). Comparisons of diacetylmorphine supplemented with medium-dose oral methadone relative to other treatments showed similar patterns as diacetylmorphine supplemented with low-dose oral methadone; however, most associations were non-significant.

### Other outcomes

Estimated MDs in the number of days of opioid and cocaine use across the two networks are presented in Additional file 1 - S5-S6 Tables and Additional file 1 - S7-S8 Tables, respectively. Due to limited data on the criminalized activity outcome, only ORs for the treatment network defined at the medication level are presented (Additional file 1 - S9 Table).

### Days of opioid use in the past month

**Fig. 4:**
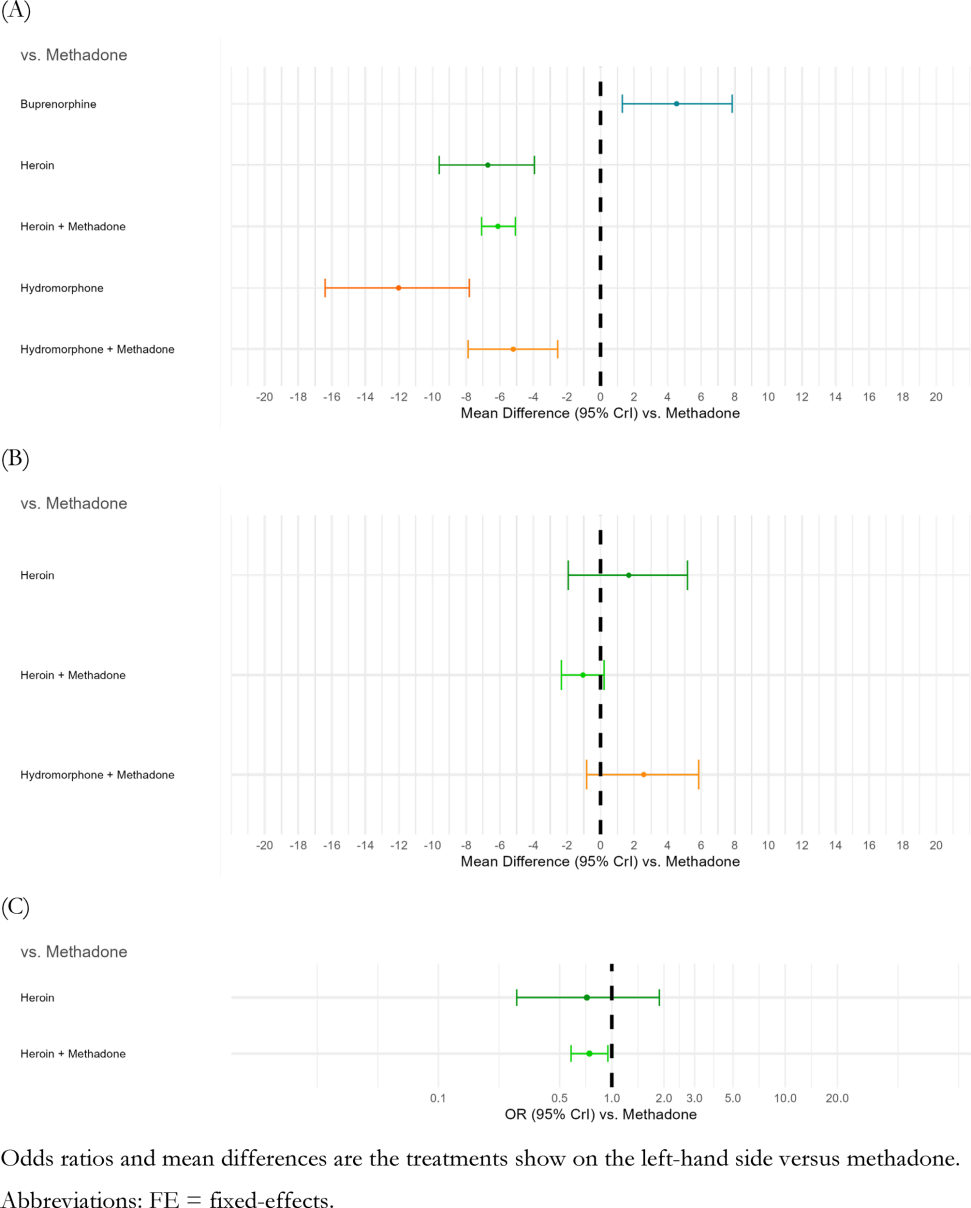
Comparative effects (odds ratios or mean difference, and 95% credible intervals) of days of opioid use (panel A), days of cocaine use (panel B), and proportion involved in criminalized activity (panel C) at 3 to 12 months of follow-up

In the treatment network defined at the medication level (Fig. 4A, Additional file 1 - S5 Table), methadone was only statistically favoured against sublingual buprenorphine (i.e., fewer days of opioid use) (MD: −4.53 days; 95% CrI: −7.84, −1.30). Sublingual buprenorphine was not favoured in any comparisons. Injectable hydromorphone supplemented with optional oral methadone had statistically favourable mean differences relative to methadone (MD: −5.20 days; 95% CrI: −7.89, −2.55) and sublingual buprenorphine (MD: −9.77 days; 95% CrI: −14.00, −5.54). Effect sizes were similar in comparisons of diacetylmorphine supplemented with optional oral methadone against methadone (MD: −6.11 days; 95% CrI: −7.08, −5.07) and sublingual buprenorphine (MD: −10.63 days; 95% CrI: −14.11, −7.25). The overall pattern of estimate directions was similar when treatments were separated by strength (Additional file 1 - S6 Table).

### Days of cocaine use in the past month

Days of cocaine use were similar across treatments in the network defined at the medication level (Fig. 4B), except diacetylmorphine supplemented with optional oral methadone had statistically fewer days relative to injectable hydromorphone supplemented with methadone (MD [95%CrI] −3.61 days [−6.68, −0.45]) (Additional file 1 - S7 Table). Statistically significant differences persisted when the network was defined at the dosage strength level (Additional file 1 - S8 Table): favouring diacetylmorphine supplemented with low- and medium-dose oral methadone over injectable hydromorphone supplemented with low-dose oral methadone (MD [95%CrI] −3.56 days [−6.68, −0.41]; MD [95%CrI] −7.40 days [−12.13, −2.57], respectively). Days of cocaine use for injectable hydromorphone supplemented with low-dose oral methadone were not significantly different from medium- and high-dose methadone, and point estimates favoured the latter comparators (MD [95%CrI] −3.57 days [−8.72, 1.68]; MD [95%CrI] −3.06 days [−6.28, 0.24], respectively).

### Proportion participating in criminalized activities

Only three treatment nodes were defined for this outcome, even with treatments defined by medication only (methadone, diacetylmorphine supplemented with oral methadone, and diacetylmorphine) (Additional file 1 - S9 Table). The only statistically significant OR was between diacetylmorphine supplemented with optional oral methadone and methadone (Fig. 4C), which showed a smaller proportion of participation in criminalized activities associated with diacetylmorphine supplemented with optional oral methadone (OR [95%CrI] 0.74 [0.58, 0.95]).

### Sensitivity analyses

The treatment network was redefined by separating methadone and sublingual buprenorphine nodes based on whether doses were personalized or fixed (see Additional file 1 - S2 Text, Additional file 1 - S2 Fig). Point estimates showed individually titrated methadone and sublingual buprenorphine had non-significantly better retention outcomes compared to their respective fixed dosage counterparts. (Additional file 1 - S3 Fig, Additional file 1 - S10-S12 Tables). Individually titrated versions of either drug also had non-significantly better retention compared to fixed-dose versions of the other drug. Results of injectable hydromorphone supplemented with optional oral methadone compared to all other interventions was generally consistent with the other two networks, but ORs were non-significant: it had favourable ORs compared to conventional OATs and comparable ORs with diacetylmorphine supplemented with oral methadone.

Additional sensitivity analyses found that estimates from the base case do not change appreciably when the evidence base was limited to studies that only include patients with OAT medication experience, to studies that have 6 months of follow-up, or to studies that do not provide any additional benefit for participation (Additional file 1 - S4 Fig).

## Discussion

OAT is an effective intervention for OUD, shown to outperform non-medication approaches in reducing opioid use, improving retention, and lowering the risk of overdose and death [12, 73]. In addition to these core outcomes, it facilitates patients engaging with healthcare, securing housing, and regain stability in their daily lives, making it foundational to both clinical care and broader recovery [21, 36, 74]. Among available OAT options, oral methadone and buprenorphine are the most widely used [75]. Methadone is a full opioid agonist with strong evidence for effectiveness and flexible dose titration, but typically requires daily witnessed dosing and carries a higher risk of overdose [13]. Buprenorphine, a partial agonist, has a favourable safety profile and is available in take-home formulations, but may be less effective for individuals with high opioid tolerance [13]. People with OUD who continue using injectable street opioids require alternative OATs to increase treatment accessibility and continuity of care [74]. Injectable hydromorphone and diacetylmorphine, are relevant alternative short-acting full agonists, but are currently less commonly used due to regulatory and logistical barriers [76]. Based on the results of this study, it was possible to ascertain the comparative effectiveness of different OAT medications. In addition to synthesizing the comparative effectiveness of conventional OATs, the results of this network meta-analysis also provide new evidence regarding the comparative effectiveness of injectable OAT (iOAT) that has only been compared with methadone in RCTs. For example, our results show that injectable hydromorphone supplemented with oral methadone (n_studies_ = 1) is associated with greater retention in assigned treatment by the end of the trial compared to high-dose methadone, low-dose methadone, medium-dose sublingual buprenorphine, low-dose sublingual buprenorphine, buprenorphine/naloxone, and depot injectable naltrexone.

The results of our network meta-analysis that relate to comparisons among conventional OATs (i.e., methadone and buprenorphine) are consistent with existing traditional meta-analyses and network meta-analyses [13, 30, 77, 78]. In a meta-analysis examining methadone strength and flexibility on retention, Bao et al. [77] found methadone doses ≥ 60 mg/day had favourable outcomes relative to methadone < 60 mg/day, and that individually-titrated dosing had greater retention compared to fixed-dosing. We found that higher strengths of methadone (high- and medium-dose) had favourable retention compared to low-dose methadone, and that individually-titrated methadone was associated with greater retention compared to fixed-dose methadone – though our estimates were non-significant. Barnett et al.’s [78] meta-analysis showed patients treated with 8-12 mg/day of buprenorphine (which would be considered “medium” dose in our study) were at greater risk of discontinuation relative to patients who received 50 to 80 mg/day of methadone (“medium”), but at lesser risk compared to 20 to 35 mg/day of methadone (“low”). The directions of these associations were also consistent with our findings. Mattick et al.’s [13] meta-analysis found flexible-dose methadone was favoured over flexible-dose buprenorphine; low-dose methadone was favoured over low-dose buprenorphine; and, no difference among medium-dose and high-dose comparisons in terms of retention. Our results for medications separated by strength are in-line with Mattick et al. [13]; whereas, our estimate for flexible-dosing was non-significant but point in the same direction. Finally, in a recent NMA comparing conventional OAT, Lim et al. [30] showed methadone had better retention outcomes relative to buprenorphine and naltrexone; however, medications were not distinguished by their strength or dosing schedules. These associations were non-statistically significant in our NMA; however, point estimates are in the same direction and of a similar magnitude as in Lim et al.’s study [30].

Our findings provide new evidence to help inform research on iOATs with short-acting medications, and may help address knowledge-related barriers to its acceptance and implementation. This is particularly important given that iOAT engages individuals with complex needs, often those for whom standard treatments have not been effective. Qualitative studies of iOAT clients and providers underscore that autonomy in choosing medication type and formulation is a valued feature of care, and for clients, a key factor in remaining engaged with treatment [79, 80]. Relatedly, results of this NMA might also help bolster the evidence base for iOAT in existing clinical guidelines. For example, Australia’s first study of supervised injectable opioid treatment is underway to determine if iOAT will be feasible and acceptable [81]. Because hydromorphone is not registered for treatment of OUD in Australia [81], the evidence generated from their trial, along with the evidence generated from this NMA on injectable hydromorphone (n_studies_ = 2) will be necessary when applying for new indications. As another example, one set of guidelines in Canada rates the quality of the current evidence for the selection between injectable hydromorphone and injectable diacetylmorphine as low based on the availability of only one RCT [28, 82]. Current guidelines suggest that either medications are acceptable and may be considered reasonable choices.

Our study has numerous strengths and limitations. First, our systematic literature review comprehensively updated the evidence base established in multiple previously published systematic reviews. Second, using a meta-regression enabled us to adjust for some differences between the study populations. Third, we conducted a number of sensitivity analyses that corroborate the main findings. For example, we accounted for the evolution of conventional OAT dosing by analyzing fixed-doses (more typical of earlier regimens) and personalized-doses (introduced later), varied follow-up duration by examining studies that have similar follow-ups, trial quality by excluding trials that offered extra benefits; and importantly, examining trials by whether prior OAT experience was an inclusion criterion or not.

Limitations include focusing the systematic literature review to English-language studies and on a population that primarily uses injectable street opioids. We recognize that this resulted in excluding studies that examined more diverse populations of people with OUD, such as the EXPO study which reported that less than 10% of the sample used injectables in the past 28 days at baseline and less than 50% used opioids in the same period [83]. The differences in definitions and timings of outcomes across studies also presented challenges with comparability. Some trials included a mix of individuals with and without prior OAT treatment, where outcomes were not reported separately. Based on our sensitivity analyses restricting to studies with similar follow-up durations, dosing regimens, and prior OAT treatment, these limitations increased the uncertainty in the estimates but did not generally change the direction of the findings overall. Finally, a number of the secondary outcomes of interest only had sparse data, and should be interpreted with caution as not all treatments could be incorporated into the analyses.

We identified a small number of study protocols for relevant trials that have yet to be completed. As such, this study should be viewed as a living NMA that warrants updating as new evidence becomes available, especially as novel treatments that have not been addressed by this current NMA emerge. Where possible, future and ongoing RCTs should report stratified results to enable subgroup analyses to be performed. Our analysis can support decision makers to make informed decisions when prioritizing resources for future trials. For example, our results suggest that it is unlikely iOAT will be inferior to conventional OAT in individuals with prior treatment experience. Robust evidence on SROM remains to be generated, as also found in Lim et al. [30]. This is particularly relevant considering that there is extensive clinical data on the positive uptake of this medication across settings, however in many settings it is prescribed off-label, limiting its prescription as OAT, due to lack of evidence.

In summary, our study finds methadone has favourable or comparable treatment retention to buprenorphine depending on the strength of the medications being compared. Sublingual buprenorphine showed inferior or comparable retention to other OAT options. Diacetylmorphine supplemented with oral methadone and injectable hydromorphone supplemented with oral methadone showed favourable or comparable retention to methadone and sublingual buprenorphine, depending on the strength of the conventional OAT being co-prescribed or being compared to. Limited comparisons for secondary outcomes suggest similar directions in the associations for days of opioid use: favouring methadone over sublingual buprenorphine and favouring diacetylmorphine supplemented with oral methadone and injectable hydromorphone supplemented with methadone over methadone and sublingual buprenorphine. Trials where all participants had prior OAT experience and trials where there was a mix of prior OAT experience did not differ, however a lack of subgroup analyses in the latter type of trials preclude additional conclusions.

## Supplementary Information

Below is the link to the electronic supplementary material.

Supplementary Material 1

## Data Availability

All data analysed during this study are cited in this published article and its supplementary information files. The datasets generated from published literature and analysed during the current study are available from the corresponding author on reasonable request.
