# Supplementary material for: Oral and injectable opioid agonist treatments for people who use street opioids: a systematic literature review and network meta-analysis
Source: BMC Public Health. 2025 Aug 30;25:2974. doi: 10.1186/s12889-025-24365-w (PMC12398115; doi:10.1186/s12889-025-24365-w)
Supplement: Supplementary file 1 — Supplementary Material 1 [file 12889_2025_24365_MOESM1_ESM.docx]

S1 Table. Risk of bias assessment of included studies assessed using Cochrane Collaboration’s tool for assessing risk bias.

| Study ID | Sequence generation | Deviation from intended intervention | Missing Outcome data | Bias in measurement | Selection of reported result | Overall |
| --- | --- | --- | --- | --- | --- | --- |
| Perneger et al. 1998 | Some concerns | Some concerns | Low | High | Some concerns | High |
| van den Brink et al. 2003 | Low | Some concerns | Low | Low | Some concerns | Some concerns |
| March et al. 2006 | Low | Low | Some concerns | Low | Some concerns | Some concerns |
| Haasen et al. 2007 | Low | Low | Low | Low | Low | Low |
| Oviedo-Joekes et al. 2009 | Low | Low | Low | Low | Low | Low |
| Strang et al. 2010 | Low | Low | Low | Low | Low | Low |
| Demaret et al. 2015 | Low | Low | Low | Low | Some concerns | Some concerns |
| Oviedo-Joekes et al. 2016 | Low | Low | Low | Low | Low | Low |
| Potteret al. 2013 | Some concerns | Some concerns | High | Some concerns | Low | High |
| Mattick et al. 2003 | Low | Low | Low | Low | Low | Low |
| Soyka et al. 2008 | Low | Low | High | Low | Low | High |
| Lintzeris et al. 2004a | Low | Low | High | Low | Some concerns | High |
| Ling et al. 1996 | Low | Low | Low | Low | Some concerns | Some concerns |
| Neri et al. 2005 | Low | Low | Low | Low | Some concerns | Some concerns |
| Pani et al. 2000 | Low | Low | High | Low | Some concerns | High |
| Fischer et al. 1999 | Low | Some concerns | Low | Low | Some concerns | Some concerns |
| Schottenfeld et al. 2005a | Some concerns | Some concerns | Some concerns | Low | Some concerns | Some concerns |
| Jutras-Aswad et al. 2022 | Low | Some concerns | Low | Low | Low | Some concerns |
| Oliveto et al. 1999a | Some concerns | Low | High | Low | Some concerns | High |
| Opheim et al. 2021 | Low | Low | Low | Low | Low | Low |
| Lee et al. 2018 | Low | Low | Low | Low | Low | Low |
| Johnson et al. 1992 | Some concerns | Some concerns | Low | Low | Some concerns | Some concerns |
| Schottenfeld et al. 1997a | Low | Low | Low | Low | Some concerns | Some concerns |
| Kosten et al. 1993 | Some concerns | Low | High | Low | Some concerns | High |

S2 Table. Definition of treatment retention in included studies.

| Study ID | Retention definition |
| --- | --- |
| Perneger et al. 1998 | Completed study by follow-up at 6 months |
| van den Brink et al. 2003 | Completed treatment administered on the 12^th^ month |
| March et al. 2006 | Completed treatment administered on the 9^th^ month |
| Haasen et al. 2007 | Completed 12-months of treatment |
| Oviedo-Joekes et al. 2009 | Retained in addiction treatment at 12 months: either receipt of the study medication on at least 10 of the 14 days before the 12-month assessment, or confirmation of retention in any other treatment program or abstinence from opioids during this interval  Per protocol data were also available |
| Strang et al. 2010 | Remained in assigned treatment at 26 weeks (6 months) |
| Demaret et al. 2015 | Completed the assigned treatment at the 12^th^ month assessment |
| Oviedo-Joekes et al. 2016 | Per protocol: received the assigned injectable medications at least 20 days in the month before the 6-month assessment. |
| Potteret al. 2013 | Completed the 24 weeks medication phase |
| Mattick et al. 2003 | Completed the study and attended the assessment at 13 weeks |
| Soyka et al. 2008 | Completed the 26-week study, and substitutions from one treatment to another treatment was allowed |
| Lintzeris et al. 2004a | Remained in substitution treatment at 6 months. |
| Ling et al. 1996 | Completed 26 weeks of treatment and remained active in the study |
| Neri et al. 2005 | Completed treatment at 6 months |
| Pani et al. 2000 | Retention in respective treatment after 6 months |
| Fischer et al. 1999 | Completed 24-week study period |
| Schottenfeld et al. 2005a | Completed 24 weeks of treatment |
| Jutras-Aswad et al. 2022 | Having both an active prescription and a positive  urine drug screen result for the assigned OAT at week  24  Switching OAT during the trial were not considered retained in the assigned treatment, but retention on any OAT was also reported |
| Oliveto et al. 1999a | Completed study and 13 weeks of treatment |
| Opheim et al. 2021 | Remained in the study at follow-up |
| Lee et al. 2018 | Completed the 24-week treatment plan |
| Johnson et al. 1992 | Active in the study at the end of 25 weeks. Participants were considered active until the third consecutive missed medication administration visit |
| Schottenfeld et al. 1997a | Retained in treatment at 24-weeks |
| Kosten et al. 1993 | Retained in study and retained on assigned medication by 24 weeks |

S3 Table. Odds Ratio of Retention at any follow-up (3 to 12 months) from random-effects NMA with meta-regression adjustment for prior treatment experience and with treatments defined by medication.

| **Methadone** | 1.29  (0.60, 2.63) | 2.22  (0.85, 5.48) | **0.50  (0.27, 0.94)** | **0.12  (0.02, 0.63)** | 0.48  (0.11, 2.15) | 2.04  (0.53, 7.12) | 0.46  (0.16, 1.31) |
| --- | --- | --- | --- | --- | --- | --- | --- |
| 0.77  (0.38, 1.66) | **Buprenorphine** | 1.72  (0.66, 4.53) | 0.39  (0.15, 1.05) | **0.09  (0.01, 0.58)** | 0.37  (0.07, 2.00) | 1.58  (0.42, 5.83) | 0.36  (0.10, 1.34) |
| 0.45  (0.18, 1.17) | 0.58  (0.22, 1.52) | **Buprenorphine_plus_Naloxone** | **0.22  (0.08, 0.72)** | **0.06  (0.01, 0.37)** | 0.21  (0.04, 1.30) | 0.92  (0.36, 2.22) | **0.21  (0.05, 0.86)** |
| **2.01  (1.07, 3.71)** | 2.59  (0.95, 6.61) | **4.45  (1.39, 13.19)** | **Heroin_plus_**  **Methadone** | 0.24  (0.03, 1.40) | 0.95  (0.24, 3.74) | 4.09  (0.93, 16.17) | 0.92  (0.27, 3.11) |
| **8.19  (1.58, 53.29)** | **10.58  (1.74, 77.57)** | **18.18  (2.72, 144.83)** | 4.10  (0.72, 28.90) | **Hydromorphone** | 3.91  (0.43, 42.68) | **16.77  (1.98, 155.77)** | 3.76  (0.73, 23.98) |
| 2.10  (0.46, 9.31) | 2.71  (0.50, 14.01) | 4.69  (0.77, 26.39) | 1.05  (0.27, 4.10) | 0.26  (0.02, 2.30) | **Hydromorphone_plus_Methadone** | 4.31  (0.58, 29.63) | 0.96  (0.15, 5.89) |
| 0.49  (0.14, 1.87) | 0.63  (0.17, 2.38) | 1.08  (0.45, 2.76) | 0.24  (0.06, 1.08) | **0.06  (0.01, 0.51)** | 0.23  (0.03, 1.73) | **Naltrexone** | 0.22  (0.04, 1.24) |
| 2.17  (0.76, 6.43) | 2.80  (0.75, 10.29) | **4.84  (1.16, 19.73)** | 1.08  (0.32, 3.76) | 0.27  (0.04, 1.37) | 1.04  (0.17, 6.56) | 4.45  (0.81, 23.08) | **Heroin** |

Each cell is the comparative effect (odds ratio and 95% credible interval) of the row treatment versus the column treatment. Bolded values are statistically significant at *p*<0.05.

S4 Table. Odds Ratio of Retention at any follow-up (3 to 12 months) from random-effects NMA with meta-regression adjustment for prior treatment experience and with treatments defined by medication and dosage strength where possible.

| **Methadone_H** | 0.59  (0.06, 4.88) | **3.66  (1.39, 9.06)** | 1.36  (0.63, 2.71) | **2.39  (1.04, 4.72)** | **0.27  (0.12, 0.55)** | 0.93  (0.40, 1.99) | **0.13  (0.02, 0.56)** | **0.26  (0.06, 0.96)** | 2.04  (0.23, 24.42) | 1.60  (0.75, 3.25) | 0.99  (0.48, 1.89) | 2.32  (0.72, 5.83) | 0.54  (0.20, 1.48) |
| --- | --- | --- | --- | --- | --- | --- | --- | --- | --- | --- | --- | --- | --- |
| 1.69  (0.20, 18.00) | **Buprenorphine_H** | 6.20  (0.58, 76.87) | 2.31  (0.24, 26.74) | 3.99  (0.41, 45.79) | 0.45  (0.05, 5.41) | 1.57  (0.15, 19.07) | 0.22  (0.01, 3.55) | 0.43  (0.03, 6.28) | 3.63  (0.16, 99.89) | 2.69  (0.27, 31.59) | 1.66  (0.17, 19.22) | 3.84  (0.35, 48.73) | 0.91  (0.09, 11.87) |
| **0.27  (0.11, 0.72)** | 0.16  (0.01, 1.71) | **Buprenorphine_L** | **0.37  (0.16, 0.85)** | 0.65  (0.21, 1.87) | **0.07  (0.02, 0.22)** | **0.25  (0.09, 0.71)** | **0.04  (0.01, 0.21)** | **0.07  (0.01, 0.34)** | 0.55  (0.05, 7.83) | **0.43  (0.20, 0.96)** | **0.27  (0.12, 0.58)** | 0.63  (0.16, 2.25) | **0.15  (0.04, 0.60)** |
| 0.73  (0.37, 1.59) | 0.43  (0.04, 4.24) | **2.68  (1.17, 6.33)** | **Buprenorphine_M** | 1.74  (0.72, 4.08) | **0.20  (0.08, 0.49)** | 0.68  (0.29, 1.57) | **0.10  (0.02, 0.51)** | **0.19  (0.04, 0.79)** | 1.50  (0.16, 19.75) | 1.18  (0.60, 2.38) | 0.72  (0.50, 1.07) | 1.69  (0.53, 5.00) | 0.39  (0.12, 1.47) |
| **0.42  (0.21, 0.97)** | 0.25  (0.02, 2.42) | 1.54  (0.53, 4.83) | 0.58  (0.25, 1.38) | **Buprenorphine_M_plus_Naloxone** | **0.11  (0.04, 0.32)** | 0.39  (0.14, 1.10) | **0.06  (0.01, 0.30)** | **0.11  (0.02, 0.49)** | 0.86  (0.09, 11.45) | 0.67  (0.26, 1.90) | 0.42  (0.18, 1.02) | 0.97  (0.46, 1.89) | **0.23  (0.07, 0.85)** |
| **3.67  (1.83, 8.35)** | 2.21  (0.18, 21.54) | **13.55  (4.51, 42.52)** | **5.07  (2.03, 12.47)** | **8.80  (3.11, 23.96)** | **Heroin_plus_Methadone_L** | **3.44  (1.25, 9.66)** | 0.49  (0.08, 2.59) | 0.95  (0.31, 2.95) | 7.60  (0.79, 99.94) | **5.88  (2.34, 16.33)** | **3.66  (1.57, 8.82)** | **8.54  (2.30, 27.93)** | 1.98  (0.60, 7.52) |
| 1.08  (0.50, 2.49) | 0.64  (0.05, 6.57) | **3.94  (1.41, 11.36)** | 1.46  (0.64, 3.42) | 2.55  (0.91, 7.02) | **0.29  (0.10, 0.80)** | **Heroin_plus_Methadone_M** | **0.14  (0.02, 0.77)** | 0.27  (0.06, 1.28) | 2.20  (0.23, 29.78) | 1.72  (0.70, 4.48) | 1.06  (0.50, 2.34) | 2.47  (0.67, 8.26) | 0.57  (0.17, 2.19) |
| **7.50  (1.77, 42.23)** | 4.50  (0.28, 67.28) | **27.52  (4.82, 192.48)** | **10.21  (1.95, 64.72)** | **17.86  (3.32, 109.66)** | 2.03  (0.39, 12.66) | **6.95  (1.30, 45.11)** | **Hydromorphone** | 1.96  (0.26, 16.02) | **15.68  (1.33, 263.52)** | **12.17  (2.35, 77.24)** | **7.38  (1.46, 46.84)** | **17.17  (2.67, 118.22)** | 4.05  (0.93, 22.71) |
| **3.88  (1.05, 15.59)** | 2.32  (0.16, 29.92) | **14.21  (2.98, 69.52)** | **5.30  (1.26, 22.28)** | **9.23  (2.04, 41.05)** | 1.05  (0.34, 3.18) | 3.65  (0.78, 16.43) | 0.51  (0.06, 3.88) | **Hydromorphone_plus_Methadone_L** | 7.99  (0.65, 137.99) | **6.25  (1.49, 27.50)** | 3.83  (0.95, 15.70) | **8.98  (1.62, 43.95)** | 2.08  (0.41, 11.85) |
| 0.49  (0.04, 4.31) | 0.28  (0.01, 6.29) | 1.80  (0.13, 18.57) | 0.67  (0.05, 6.38) | 1.16  (0.09, 11.05) | 0.13  (0.01, 1.27) | 0.45  (0.03, 4.41) | **0.06  (0.00, 0.75)** | 0.13  (0.01, 1.53) | **Methadone** | 0.79  (0.06, 7.62) | 0.49  (0.04, 4.55) | 1.13  (0.07, 11.76) | 0.27  (0.03, 1.79) |
| 0.63  (0.31, 1.34) | 0.37  (0.03, 3.64) | **2.30  (1.05, 4.91)** | 0.85  (0.42, 1.68) | 1.49  (0.53, 3.81) | **0.17  (0.06, 0.43)** | 0.58  (0.22, 1.43) | **0.08  (0.01, 0.42)** | **0.16  (0.04, 0.67)** | 1.27  (0.13, 16.84) | **Methadone_L** | 0.62  (0.32, 1.16) | 1.45  (0.39, 4.51) | 0.33  (0.10, 1.20) |
| 1.01  (0.53, 2.08) | 0.60  (0.05, 5.84) | **3.70  (1.73, 8.14)** | 1.38  (0.93, 2.02) | 2.41  (0.98, 5.54) | **0.27  (0.11, 0.64)** | 0.95  (0.43, 2.01) | **0.14  (0.02, 0.68)** | 0.26  (0.06, 1.05) | 2.05  (0.22, 26.60) | 1.62  (0.86, 3.15) | **Methadone_M** | 2.33  (0.72, 6.70) | 0.54  (0.17, 1.95) |
| 0.43  (0.17, 1.38) | 0.26  (0.02, 2.86) | 1.59  (0.44, 6.39) | 0.59  (0.20, 1.90) | 1.03  (0.53, 2.16) | **0.12  (0.04, 0.44)** | 0.40  (0.12, 1.49) | **0.06  (0.01, 0.38)** | **0.11  (0.02, 0.62)** | 0.88  (0.09, 13.43) | 0.69  (0.22, 2.54) | 0.43  (0.15, 1.40) | **Naltrexone** | 0.23  (0.06, 1.09) |
| 1.86  (0.68, 5.02) | 1.10  (0.08, 11.32) | **6.85  (1.67, 25.58)** | 2.55  (0.68, 8.29) | **4.44  (1.18, 14.16)** | 0.51  (0.13, 1.66) | 1.74  (0.46, 5.90) | 0.25  (0.04, 1.08) | 0.48  (0.08, 2.44) | 3.74  (0.56, 34.80) | 2.99  (0.84, 9.90) | 1.84  (0.51, 5.92) | 4.32  (0.92, 16.42) | **Heroin** |

Each cell is the comparative effect (odds ratio and 95% credible interval) of the row treatment versus the column treatment. Bolded values are statistically significant at *p*<0.05.. Abbreviations _L, _M, and _H refer to low-, medium-, and high-dose versions of the medications. For methadone: high-dose = >80mg/day, medium-dose = 40 to 80mg/day, and low-dose = <40mg/day. For sublingual buprenorphine: high-dose >24mg/day, medium-dose 8-24mg/day, and low-dose <8mg/day.

S5 Table. Mean difference in the number of days of opioid use at any follow-up (3 to 12 months) from fixed-effects NMA with treatments defined by medication.

| **Methadone** | **-4.53  (-7.84, -1.30)** | **6.11  (5.07, 7.08)** | **12.03  (7.82, 16.41)** | **5.20  (2.55, 7.89)** | **6.71  (3.94, 9.61)** |
| --- | --- | --- | --- | --- | --- |
| **4.53  (1.30, 7.84)** | **Buprenorphine** | **10.63  (7.25, 14.11)** | **16.59  (11.15, 22.02)** | **9.77  (5.54, 14.00)** | **11.29  (6.92, 15.68)** |
| **-6.11  (-7.08, -5.07)** | **-10.63  (-14.11, -7.25)** | **Heroin_plus_Methadone** | **5.92  (1.56, 10.28)** | -0.92  (-3.28, 1.63) | 0.63  (-2.35, 3.68) |
| **-12.03  (-16.41, -7.82)** | **-16.59  (-22.02, -11.15)** | **-5.92  (-10.28, -1.56)** | **Hydromorphone** | **-6.85  (-11.90, -1.82)** | **-5.30  (-9.31, -1.33)** |
| **-5.20  (-7.89, -2.55)** | **-9.77  (-14.00, -5.54)** | 0.92  (-1.63, 3.28) | **6.85  (1.82, 11.90)** | **Hydromorphone_plus_Methadone** | 1.54  (-2.29, 5.34) |
| **-6.71  (-9.61, -3.94)** | **-11.29  (-15.68, -6.92)** | -0.63  (-3.68, 2.35) | **5.30  (1.33, 9.31)** | -1.54  (-5.34, 2.29) | **Heroin** |

Each cell is the comparative effect (mean difference and 95% credible interval) of the row treatment versus the column treatment. Bolded values are statistically significant at *p*<0.05.

S6 Table. Mean difference in the number of days of opioid use at any follow-up (3 to 12 months) from fixed-effects NMA with treatments defined by medication and dosage strength where possible.

| **Methadone_H** | **-5.30  (-10.22, -0.74)** | **6.14  (4.97, 7.27)** | **3.44  (0.03, 6.84)** | **12.05  (7.71, 16.53)** | **5.27  (2.61, 8.08)** | -0.32  (-5.48, 4.85) | -1.33  (-3.80, 1.04) | **6.70  (3.86, 9.51)** |
| --- | --- | --- | --- | --- | --- | --- | --- | --- |
| **5.30  (0.74, 10.22)** | **Buprenorphine_L** | **11.43  (6.78, 16.31)** | **8.76  (3.56, 14.12)** | **17.34  (11.02, 24.05)** | **10.54  (5.35, 16.18)** | **5.09  (0.99, 9.08)** | 3.98  (-0.01, 8.08) | **12.06  (6.72, 17.57)** |
| **-6.14  (-7.27, -4.97)** | **-11.43  (-16.31, -6.78)** | **Heroin_plus_Methadone_L** | -2.68  (-6.19, 0.70) | **5.94  (1.35, 10.56)** | -0.89  (-3.29, 1.59) | **-6.43  (-11.51, -1.34)** | **-7.46  (-9.89, -5.17)** | 0.58  (-2.48, 3.48) |
| **-3.44  (-6.84, -0.03)** | **-8.76  (-14.12, -3.56)** | 2.68  (-0.70, 6.19) | **Heroin_plus_Methadone_M** | **8.62  (3.13, 14.23)** | 1.78  (-2.40, 6.06) | -3.77  (-9.44, 1.98) | **-4.81  (-8.26, -1.21)** | 3.24  (-1.11, 7.66) |
| **-12.05  (-16.53, -7.71)** | **-17.34  (-24.05, -11.02)** | **-5.94  (-10.56, -1.35)** | **-8.62  (-14.23, -3.13)** | **Hydromorphone** | **-6.79  (-11.93, -1.50)** | **-12.40  (-19.31, -5.74)** | **-13.35  (-18.57, -8.40)** | **-5.33  (-9.32, -1.39)** |
| **-5.27  (-8.08, -2.61)** | **-10.54  (-16.18, -5.35)** | 0.89  (-1.59, 3.29) | -1.78  (-6.06, 2.40) | **6.79  (1.50, 11.93)** | **Hydromorphone_plus_Methadone_L** | -5.59  (-11.31, 0.08) | **-6.57  (-10.09, -3.31)** | 1.43  (-2.39, 5.27) |
| 0.32  (-4.85, 5.48) | **-5.09  (-9.08, -0.99)** | **6.43  (1.34, 11.51)** | 3.77  (-1.98, 9.44) | **12.40  (5.74, 19.31)** | 5.59  (-0.08, 11.31) | **Methadone_L** | -1.00  (-5.44, 3.50) | **7.08  (1.10, 12.75)** |
| 1.33  (-1.04, 3.80) | -3.98  (-8.08, 0.01) | **7.46  (5.17, 9.89)** | **4.81  (1.21, 8.26)** | **13.35  (8.40, 18.57)** | **6.57  (3.31, 10.09)** | 1.00  (-3.50, 5.44) | **Methadone_M** | **8.04  (4.40, 11.72)** |
| **-6.70  (-9.51, -3.86)** | **-12.06  (-17.57, -6.72)** | -0.58  (-3.48, 2.48) | -3.24  (-7.66, 1.11) | **5.33  (1.39, 9.32)** | -1.43  (-5.27, 2.39) | **-7.08  (-12.75, -1.10)** | **-8.04  (-11.72, -4.40)** | **Heroin** |

Each cell is the comparative effect (mean difference and 95% credible interval) of the row treatment versus the column treatment. Bolded values are statistically significant at *p*<0.05.. Abbreviations _L, _M, and _H refer to low-, medium-, and high-dose versions of the medications. For methadone: high-dose = >80mg/day, medium-dose = 40 to 80mg/day, and low-dose = <40mg/day. For sublingual buprenorphine: high-dose >24mg/day, medium-dose 8-24mg/day, and low-dose <8mg/day.

S7 Table. Mean difference in the number of days of cocaine use at any follow-up (3 to 12 months) from fixed-effects NMA with treatments defined by medication.

| **Methadone** | 1.04  (-0.21, 2.33) | -2.57  (-5.85, 0.83) | -1.68  (-5.18, 1.92) |
| --- | --- | --- | --- |
| -1.04  (-2.33, 0.21) | **Heroin_plus_Methadone** | **-3.61  (-6.68, -0.45)** | -2.71  (-6.54, 0.98) |
| 2.57  (-0.83, 5.85) | **3.61  (0.45, 6.68)** | **Hydromorphone_plus_Methadone** | 0.90  (-3.91, 5.69) |
| 1.68  (-1.92, 5.18) | 2.71  (-0.98, 6.54) | -0.90  (-5.69, 3.91) | **Heroin** |

Each cell is the comparative effect (mean difference and 95% credible interval) of the row treatment versus the column treatment. Bolded values are statistically significant at *p*<0.05..

S8 Table. Mean difference in the number of days of cocaine use at any follow-up (3 to 12 months) from fixed-effects NMA with treatments defined by medication and dosage strength where possible.

| **Methadone_H** | 0.51  (-0.78, 1.76) | **4.32  (0.70, 7.86)** | -3.06  (-6.28, 0.24) | 0.50  (-3.34, 4.23) | -1.77  (-5.22, 1.80) |
| --- | --- | --- | --- | --- | --- |
| -0.51  (-1.76, 0.78) | **Heroin_plus_Methadone_L** | 3.80  (-0.03, 7.67) | **-3.56  (-6.68, -0.41)** | -0.05  (-4.12, 3.94) | -2.32  (-5.98, 1.55) |
| **-4.32  (-7.86, -0.70)** | -3.80  (-7.67, 0.03) | **Heroin_plus_Methadone_M** | **-7.40  (-12.13, -2.57)** | **-3.88  (-7.10, -0.58)** | **-6.01  (-11.15, -1.01)** |
| 3.06  (-0.24, 6.28) | **3.56  (0.41, 6.68)** | **7.40  (2.57, 12.13)** | **Hydromorphone_plus_Methadone_L** | 3.57  (-1.68, 8.72) | 1.33  (-3.40, 5.94) |
| -0.50  (-4.23, 3.34) | 0.05  (-3.94, 4.12) | **3.88  (0.58, 7.10)** | -3.57  (-8.72, 1.68) | **Methadone_M** | -2.18  (-7.31, 2.83) |
| 1.77  (-1.80, 5.22) | 2.32  (-1.55, 5.98) | **6.01  (1.01, 11.15)** | -1.33  (-5.94, 3.40) | 2.18  (-2.83, 7.31) | **Heroin** |

Each cell is the comparative effect (mean difference and 95% credible interval) of the row treatment versus the column treatment. Bolded values are statistically significant at *p*<0.05.. Abbreviations _L, _M, and _H refer to low-, medium-, and high-dose versions of the medications. For methadone: high-dose = >80mg/day, medium-dose = 40 to 80mg/day, and low-dose = <40mg/day. For sublingual buprenorphine: high-dose >24mg/day, medium-dose 8-24mg/day, and low-dose <8mg/day.

S9 Table. Odds Ratio of Participating in Illegal Activity at any follow-up (3 to 12 months) from fixed-effects NMA with treatments defined by medication.

| **Methadone** | **1.35  (1.05, 1.72)** | 1.39  (0.53, 3.54) |
| --- | --- | --- |
| **0.74  (0.58, 0.95)** | **Heroin_plus_Methadone** | 1.03  (0.40, 2.70) |
| 0.72  (0.28, 1.88) | 0.98  (0.37, 2.53) | **Heroin** |

Each cell is the comparative effect (mean difference and 95% credible interval) of the row treatment versus the column treatment. Bolded values are statistically significant at *p*<0.05.

S10 Table. Odds Ratio of Retention at any follow-up (3 to 12 months) from random-effects NMA with meta-regression adjustment for prior treatment experience and with treatments defined by medication and dose variability where possible.

| **Methadone_V** | 4.30  (0.30, 69.36) | 1.84  (0.53, 6.03) | 2.48  (0.82, 6.88) | **0.50  (0.26, 0.97)** | **0.13  (0.02, 0.72)** | 0.47  (0.10, 2.29) | 2.05  (0.17, 33.40) | 3.02  (0.22, 47.23) | 2.27  (0.51, 8.99) | 0.54  (0.14, 2.01) |
| --- | --- | --- | --- | --- | --- | --- | --- | --- | --- | --- |
| 0.23  (0.01, 3.32) | **Buprenorphine_F** | 0.42  (0.03, 4.53) | 0.58  (0.04, 7.82) | 0.12  (0.01, 1.80) | **0.03  (0.00, 0.75)** | 0.11  (0.00, 2.40) | 0.47  (0.01, 21.93) | 0.70  (0.42, 1.18) | 0.53  (0.03, 8.35) | 0.12  (0.01, 2.39) |
| 0.54  (0.17, 1.88) | 2.36  (0.22, 29.44) | **Buprenorphine_V** | 1.35  (0.41, 4.29) | 0.27  (0.07, 1.10) | **0.07  (0.01, 0.59)** | 0.26  (0.04, 1.95) | 1.11  (0.07, 22.67) | 1.66  (0.17, 19.45) | 1.24  (0.27, 5.43) | 0.29  (0.05, 1.79) |
| 0.40  (0.15, 1.21) | 1.73  (0.13, 27.94) | 0.74  (0.23, 2.42) | **Buprenorphine_V_plus_Naloxone** | **0.20  (0.06, 0.73)** | **0.05  (0.01, 0.40)** | 0.19  (0.03, 1.33) | 0.83  (0.06, 15.86) | 1.21  (0.09, 19.01) | 0.92  (0.35, 2.30) | 0.22  (0.04, 1.20) |
| **2.00  (1.03, 3.85)** | 8.61  (0.55, 152.04) | 3.67  (0.91, 14.30) | **4.96  (1.38, 16.94)** | **Heroin_plus_Methadone_V** | 0.27  (0.03, 1.61) | 0.95  (0.24, 3.89) | 4.12  (0.30, 71.26) | 6.10  (0.40, 103.10) | 4.59  (0.88, 20.76) | 1.07  (0.24, 4.63) |
| **7.47  (1.39, 53.14)** | **33.01  (1.33, 934.86)** | **13.74  (1.70, 132.55)** | **18.63  (2.50, 166.31)** | 3.73  (0.62, 29.34) | **Hydromorphone** | 3.58  (0.38, 42.96) | 15.54  (0.97, 327.94) | **23.42  (1.01, 618.74)** | **17.27  (1.80, 183.14)** | 4.01  (0.73, 27.64) |
| 2.12  (0.44, 9.66) | 9.08  (0.42, 220.03) | 3.90  (0.51, 26.43) | 5.22  (0.75, 33.09) | 1.06  (0.26, 4.24) | 0.28  (0.02, 2.65) | **Hydromorphone_plus_Methadone_V** | 4.36  (0.21, 96.17) | 6.42  (0.30, 150.80) | 4.83  (0.53, 37.37) | 1.13  (0.14, 8.25) |
| 0.49  (0.03, 6.02) | 2.11  (0.05, 90.55) | 0.90  (0.04, 13.98) | 1.21  (0.06, 17.99) | 0.24  (0.01, 3.30) | 0.06  (0.00, 1.03) | 0.23  (0.01, 4.67) | **Methadone** | 1.48  (0.03, 62.63) | 1.10  (0.05, 19.30) | 0.26  (0.02, 2.20) |
| 0.33  (0.02, 4.51) | 1.42  (0.85, 2.38) | 0.60  (0.05, 6.03) | 0.82  (0.05, 10.61) | 0.16  (0.01, 2.47) | **0.04  (0.00, 0.99)** | 0.16  (0.01, 3.28) | 0.67  (0.02, 29.88) | **Methadone_F** | 0.75  (0.04, 11.58) | 0.17  (0.01, 3.30) |
| 0.44  (0.11, 1.97) | 1.90  (0.12, 36.54) | 0.81  (0.18, 3.71) | 1.09  (0.43, 2.89) | 0.22  (0.05, 1.14) | **0.06  (0.01, 0.56)** | 0.21  (0.03, 1.90) | 0.91  (0.05, 20.20) | 1.33  (0.09, 25.18) | **Naltrexone** | 0.24  (0.03, 1.73) |
| 1.86  (0.50, 7.12) | 8.18  (0.42, 170.50) | 3.43  (0.56, 19.61) | 4.63  (0.83, 24.75) | 0.93  (0.22, 4.13) | 0.25  (0.04, 1.38) | 0.88  (0.12, 6.95) | 3.81  (0.46, 43.31) | 5.77  (0.30, 116.87) | 4.22  (0.58, 28.60) | **Heroin** |

Each cell is the comparative effect (odds ratio and 95% credible interval) of the row treatment versus the column treatment. Bolded values are statistically significant at *p*<0.05.. Abbreviations _F and _V refer to fixed and variable dosing regimens of the medications.

S11 Table. Mean difference in the number of days of opioid use at any follow-up (3 to 12 months) from fixed-effects NMA with treatments defined by medication and dose variability where possible.

| **Methadone_V** | **6.11  (5.07, 7.12)** | **11.95  (7.75, 16.25)** | **5.28  (2.69, 7.98)** | **6.70  (3.92, 9.52)** |
| --- | --- | --- | --- | --- |
| **-6.11  (-7.12, -5.07)** | **Heroin_plus_Methadone_V** | **5.84  (1.55, 10.36)** | -0.81  (-3.27, 1.69) | 0.59  (-2.42, 3.57) |
| **-11.95  (-16.25, -7.75)** | **-5.84  (-10.36, -1.55)** | **Hydromorphone** | **-6.64  (-11.87, -1.61)** | **-5.32  (-9.34, -1.29)** |
| **-5.28  (-7.98, -2.69)** | 0.81  (-1.69, 3.27) | **6.64  (1.61, 11.87)** | **Hydromorphone_plus_Methadone_V** | 1.39  (-2.37, 5.32) |
| **-6.70  (-9.52, -3.92)** | -0.59  (-3.57, 2.42) | **5.32  (1.29, 9.34)** | -1.39  (-5.32, 2.37) | **Heroin** |

Each cell is the comparative effect (mean difference and 95% credible interval) of the row treatment versus the column treatment. Bolded values are statistically significant at *p*<0.05.. Abbreviations _F and _V refer to fixed and variable dosing regimens of the medications.

S12 Table. Mean difference in the number of days of cocaine use at any follow-up (3 to 12 months) from fixed-effects NMA with treatments defined by medication and dose variability where possible.

| **Methadone_V** | 1.05  (-0.23, 2.35) | -2.54  (-5.99, 0.86) | -1.71  (-5.19, 1.81) |
| --- | --- | --- | --- |
| -1.05  (-2.35, 0.23) | **Heroin_plus_Methadone_V** | **-3.62  (-6.70, -0.42)** | -2.79  (-6.51, 0.95) |
| 2.54  (-0.86, 5.99) | **3.62  (0.42, 6.70)** | **Hydromorphone_plus_Methadone_V** | 0.84  (-3.93, 5.65) |

Each cell is the comparative effect (mean difference and 95% credible interval) of the row treatment versus the column treatment. Bolded values are statistically significant at *p*<0.05.. Abbreviations _F and _V refer to fixed and variable dosing regimens of the medications.


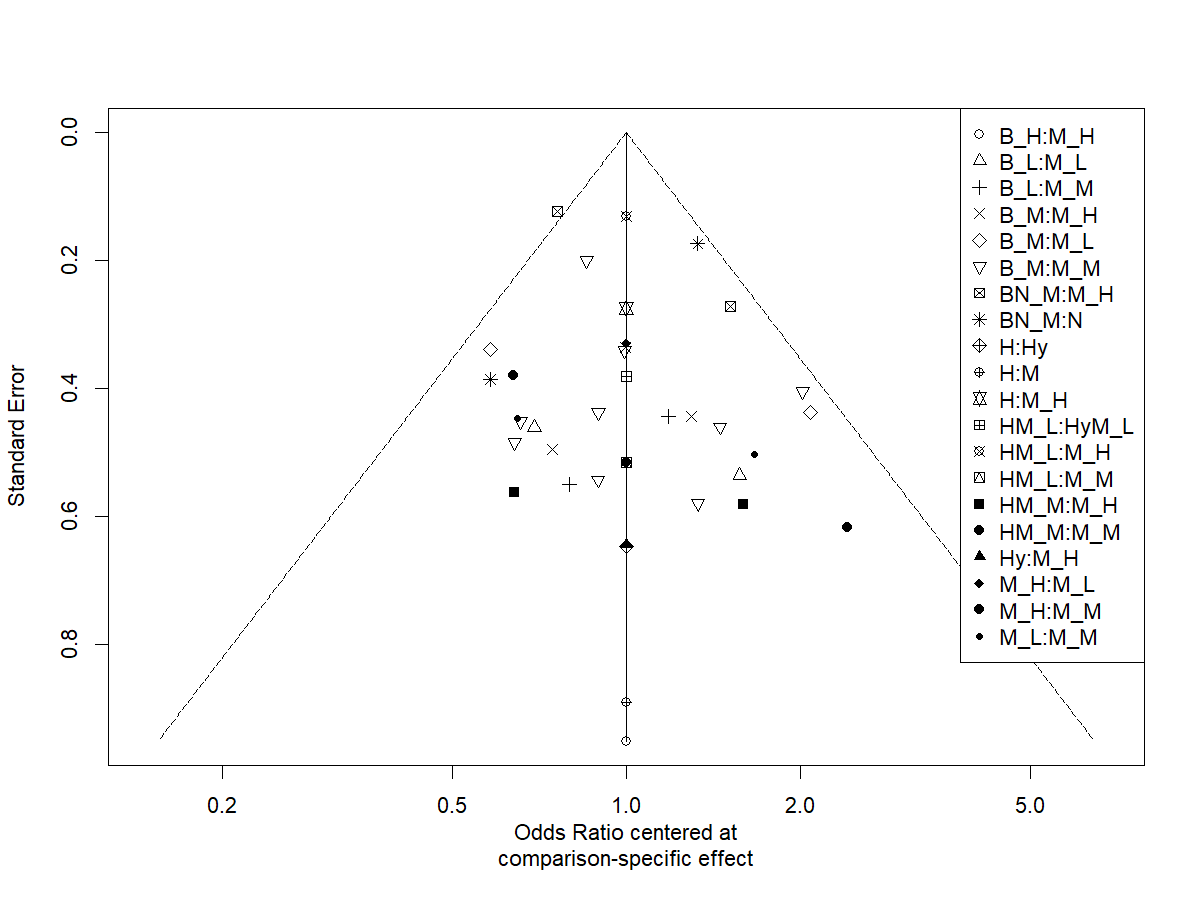

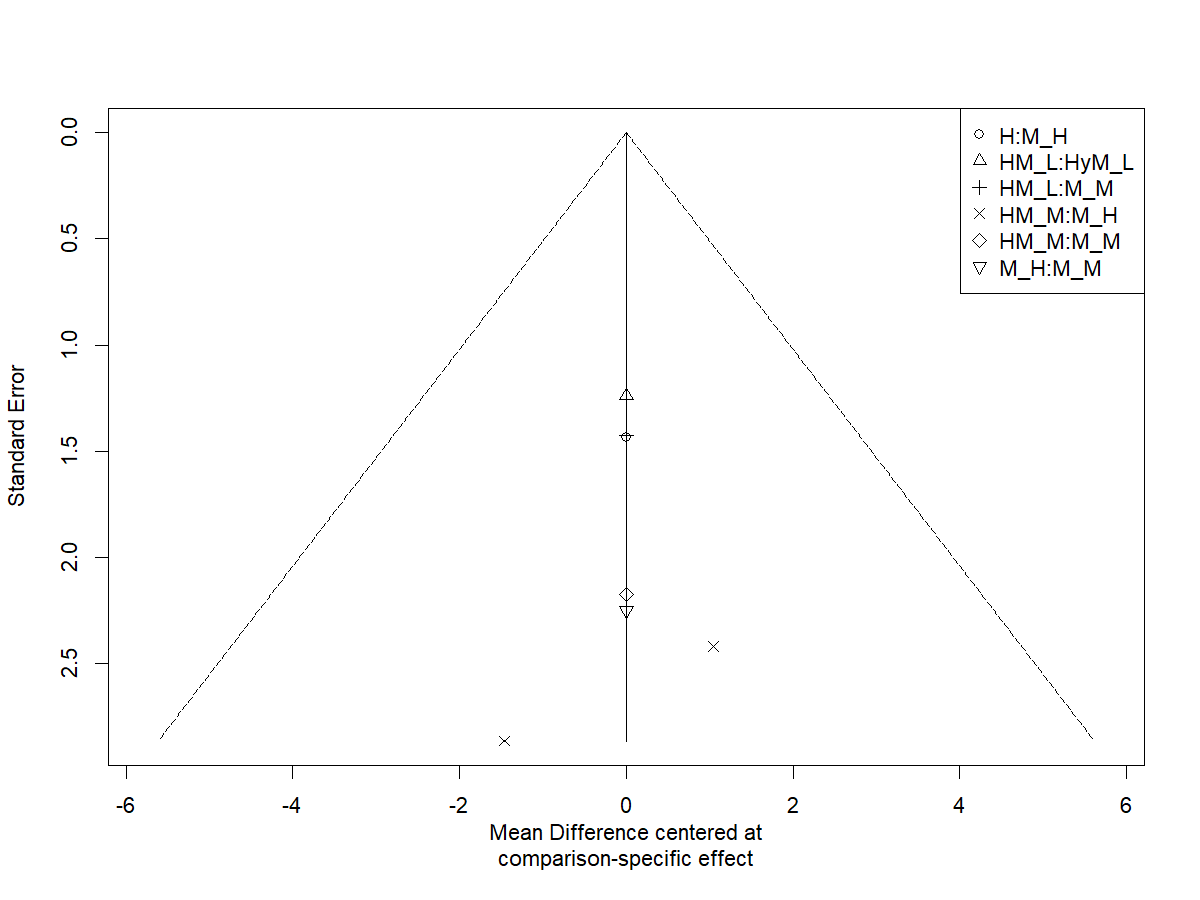


A

B


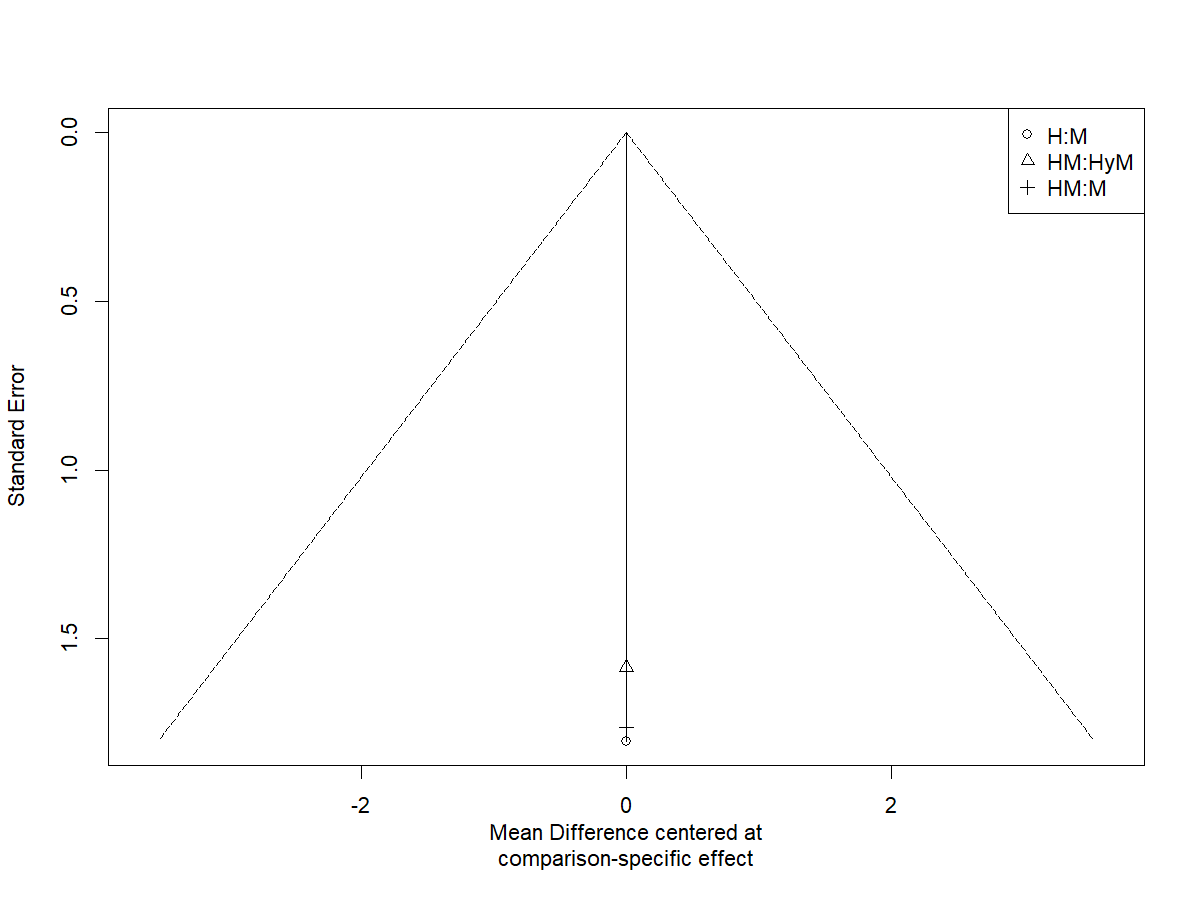

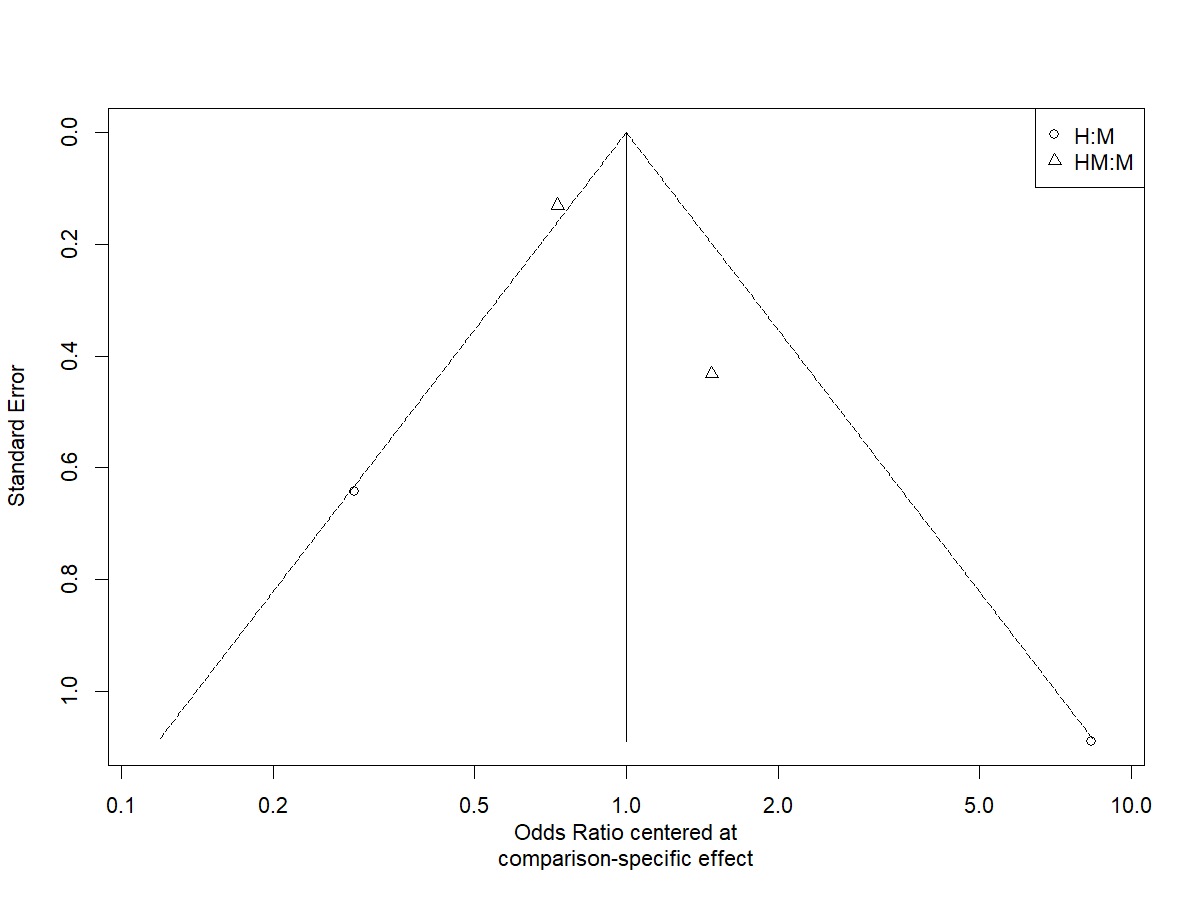


C

D

S1 Figure. Comparison-adjusted funnel plot to inspect for publication bias by outcome. Panel A = retention. Panel B = opioid use. Panel C = cocaine use. Panel D = involvement in criminalized activities. Treatment shorthands are as follows: B = buprenorphine. BN = buprenorphine and naltrexone. H = heroin. HM = heroin and methadone. Hy = hydrmorphone. HyM = hydromorphone and methadone. _L, _M, and _H refer to low-, medium-, and high-dose versions of the medications. For methadone: high-dose = >80mg/day, medium-dose = 40 to 80mg/day, and low-dose = <40mg/day. For sublingual buprenorphine: high-dose >24mg/day, medium-dose 8-24mg/day, and low-dose <8mg/day. The figure was generated using R package *netmeta (*Balduzzi et al 2023).


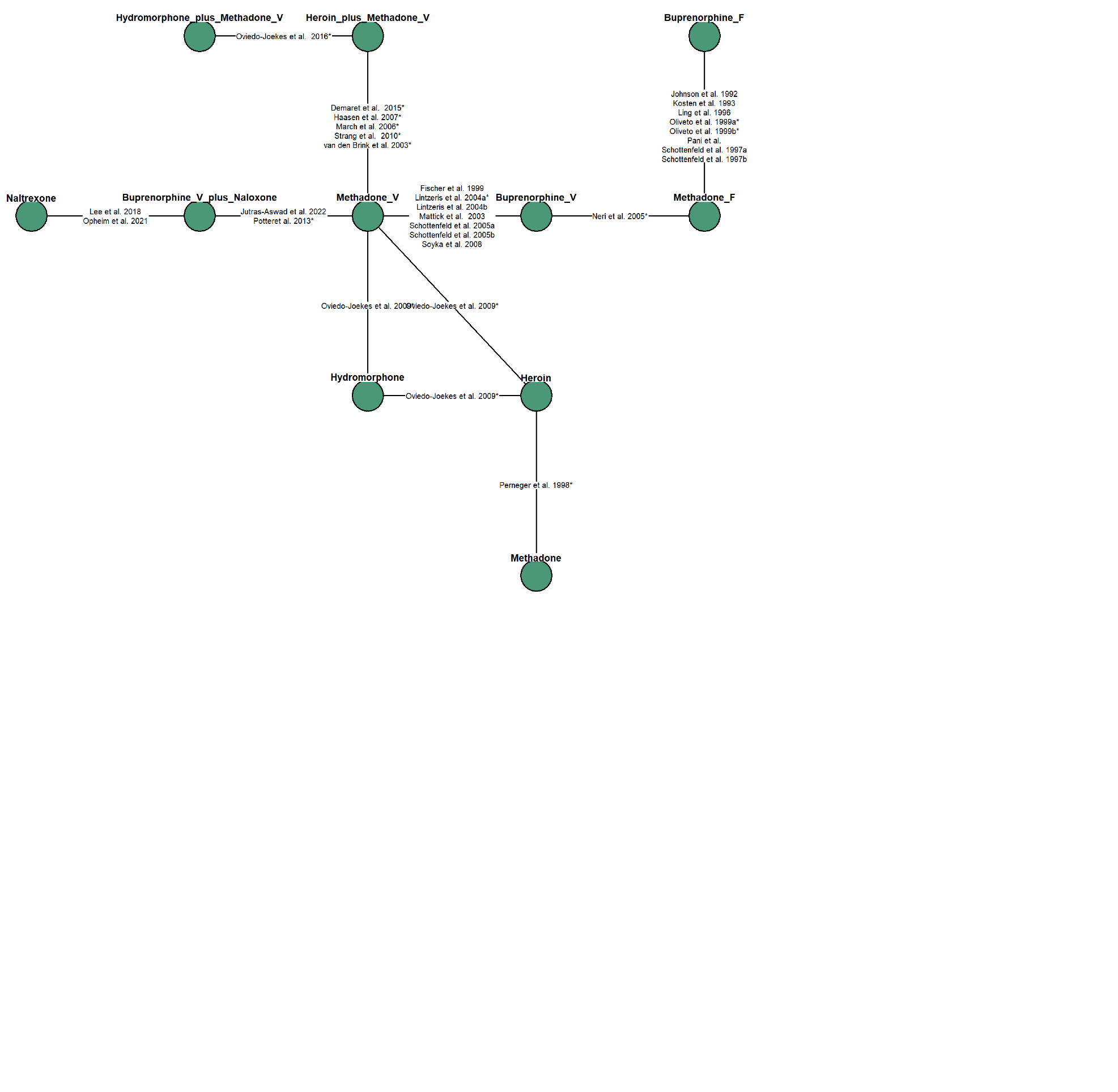


S2 Figure. Treatment network. Abbreviations: _V and _F refer to variable- and fixed-dose versions of the medications.


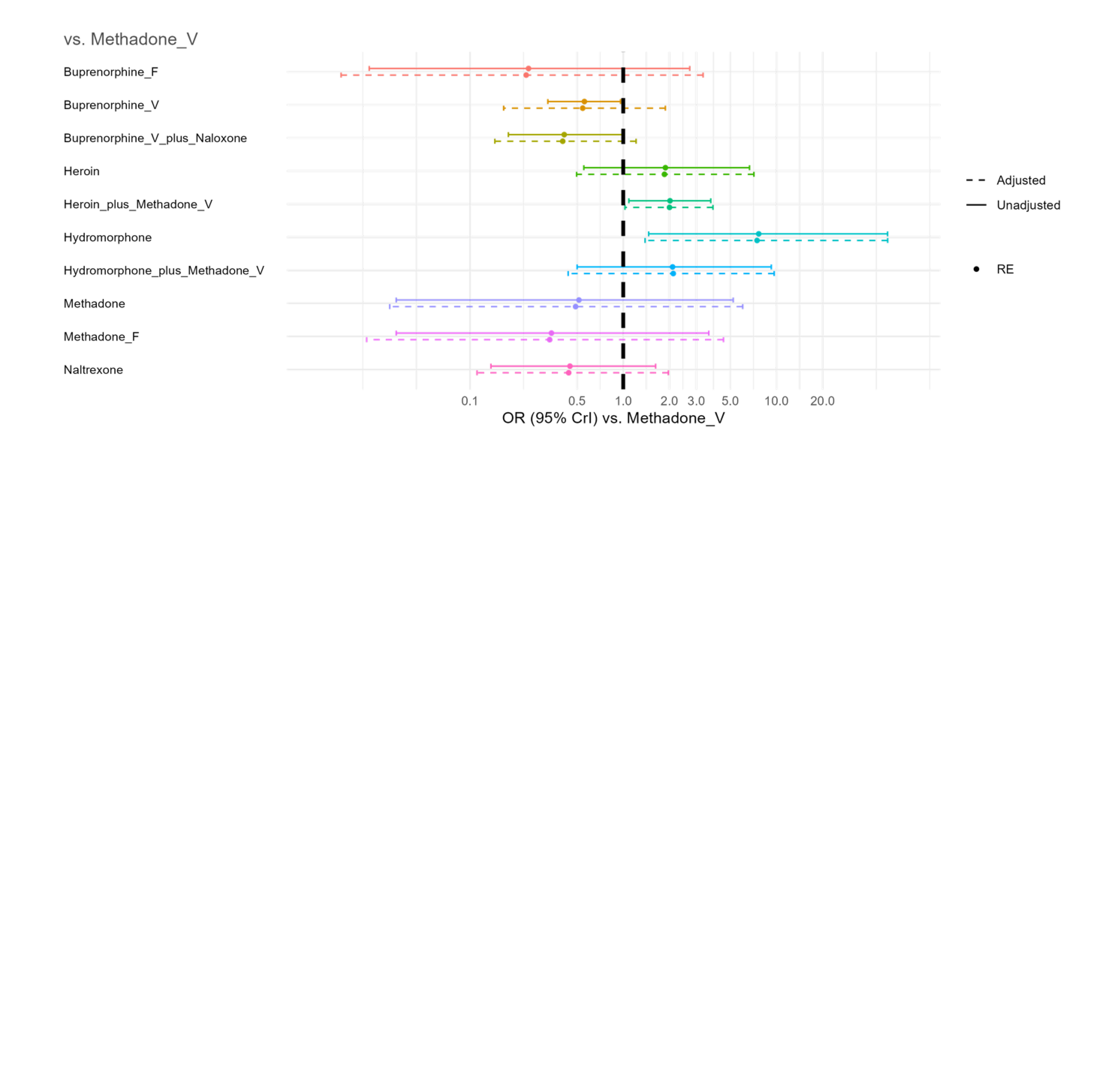


S3 Figure. Comparative effects (odds ratios and 95% credible intervals) of treatment retention at 3 to 12 months of follow-up. Odds ratios are the treatments show on the left-hand side versus methadone. Abbreviations: _V and _F refer to variable- and fixed-dose versions of the medications. RE = random-effects.


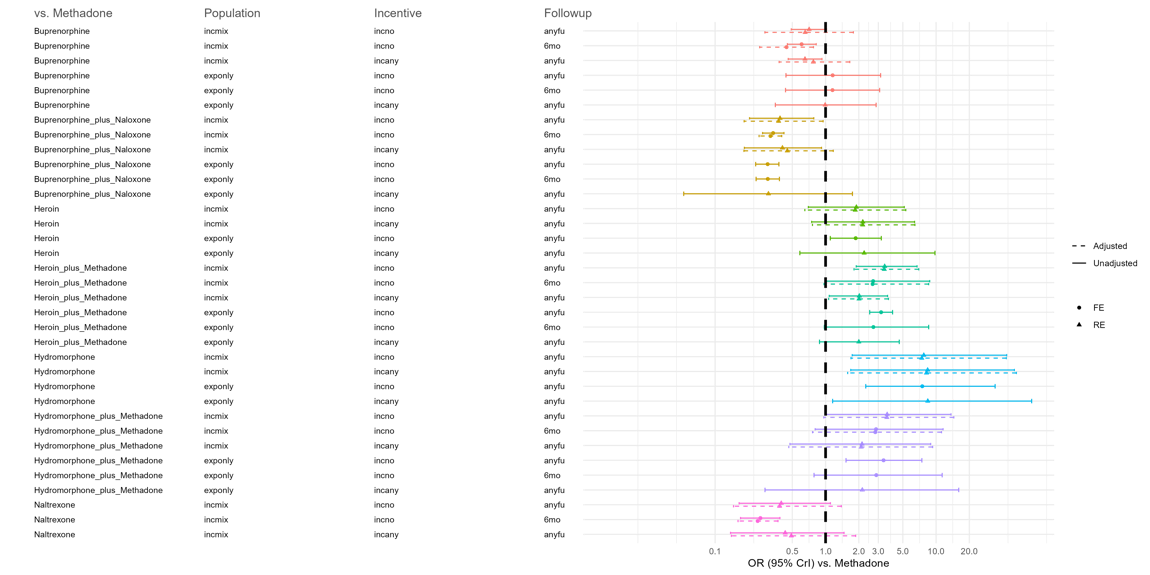


S4 Figure. Sensitivity analyses of comparative effects (odds ratios and 95% credible intervals) of treatment retention. Abbreviations: incmix = population has mixed experience of prior treatment. exponly = population has experience of prior treatment. incno = No incentives offered for trial participation. incany = Incentives were offered for trial participation. anyfu = data available at any follow-up duration (3 to 12 months). 6mo = data available at 6 months ± 0.5 months.

# S1 Text. Additional methods

Eligibility criteria in PICOS format

| Criteria | Description |
| --- | --- |
| Patient and Population | *Inclusion criteria*   - Patients with opioid use disorder and - Seeking treatment - Non-incarcerated - Non-pregnant - Majority of patients who use heroin or other injectable street opioids at baseline   *Exclusion criteria*   - Trials that examine only patients with no prior treatment experience |
| Intervention | - Methadone - Buprenorphine - Buprenorphine/naloxone - Naltrexone - Slow-release oral morphine - Diacetylmorphine - Hydromorphone |
| Comparators | - Any of the above   *Exclusion criteria*   - Comparisons of different doses, frequency, or routes of administration of the *same* medication |
| Outcomes | - Proportion of patients retained on treatment - Days of cocaine use - Days of street opioid use - Proportion of patients with involvement in criminalized activity |
| Design | Randomized control trial, minimum follow-up of 3 months |

CENTRAL Search Strategy

| 1 | exp Opioid-Related Disorders/ |
| --- | --- |
| 2 | (opioid* or opiat*).ti. |
| 3 | (opioid* or opiat*).ab. |
| 4 | (opioid* or opiat*).kw. |
| 5 | or/2-4 |
| 6 | (abus* or dependen* or disorder*).ti. |
| 7 | (abus* or dependen* or disorder*).ab. |
| 8 | (abus* or dependen* or disorder*).kw. |
| 9 | or/6-8 |
| 10 | 5 and 9 |
| 11 | ((opioid* or opiat*) and (abus* or dependen* or disorder*)).ti. |
| 12 | ((opioid* or opiat*) and (abus* or dependen* or disorder*)).ab. |
| 13 | ((opioid* or opiat*) and (abus* or dependen* or disorder*)).kw. |
| 14 | or/11-13 |
| 15 | 1 or 10 |
| 16 | 1 or 14 |
| 17 | heroin.ti. |
| 18 | heroin.ab. |
| 19 | heroin.kw. |
| 20 | or/17-19 |
| 21 | 20 or 5 |
| 22 | exp buprenorphine/ |
| 23 | exp methadone/ |
| 24 | buprenorphine.ti. |
| 25 | buprenorphine.ab. |
| 26 | buprenorphine.kw. |
| 27 | methadone.ti. |
| 28 | methadone.ab. |
| 29 | methadone.kw. |
| 30 | or/22-29 |
| 31 | 15 and 21 and 30 |
| 32 | 16 and 21 and 30 |
| 33 | limit 31 to yr="2013 - 2017" |
| 34 | limit 32 to yr="2013 - 2017" |
| 35 | limit 31 to yr="2017 - Current" |
| 36 | limit 32 to yr="2017 - Current" |

*For lines 35, 36, the search was run in October 2024

EMBASE Search Strategy

| 1 | exp addiction/ |
| --- | --- |
| 2 | exp drug abuse/ |
| 3 | ((drug or substance or opioid* or opiat*) adj3 (abuse* or addict* or depend* or disorder*)).ti. |
| 4 | ((drug or substance or opioid* or opiat*) adj3 (abuse* or addict* or depend* or disorder*)).ab. |
| 5 | or/3-4 |
| 6 | 1 or 2 or 5 |
| 7 | opioid*.ti. |
| 8 | opioid*.ab. |
| 9 | opiat*.ti. |
| 10 | opiat*.ab. |
| 11 | heroin*.ti. |
| 12 | heroin*.ab. |
| 13 | narcot*.ti. |
| 14 | narcot*.ab. |
| 15 | or/6-14 |
| 16 | exp diamorphine/ |
| 17 | or/15-16 |
| 18 | 6 and 17 |
| 19 | exp buprenorphine/ |
| 20 | buprenorphine.ti. |
| 21 | buprenorphine.ab. |
| 22 | or/19-21 |
| 23 | exp methadone/ |
| 24 | methadone.ab. |
| 25 | methadone.ti. |
| 26 | or/23-25 |
| 27 | 22 or 26 |
| 28 | exp crossover procedure/ |
| 29 | exp double blind procedure/ |
| 30 | exp single blind procedure/ |
| 31 | exp controlled clinical trial/ |
| 32 | exp clinical trial/ |
| 33 | placebo.ab. |
| 34 | placebo.ti. |
| 35 | double blind.ab. |
| 36 | double blind.ti. |
| 37 | single blind.ab. |
| 38 | single blind.ti. |
| 39 | assign*.ab. |
| 40 | assign*.ti. |
| 41 | allocat*.ab. |
| 42 | allocat*.ti. |
| 43 | volunteer*.ab. |
| 44 | volunteer*.ti. |
| 45 | random*.ab. |
| 46 | random*.ti. |
| 47 | factorial*.ab. |
| 48 | factorial*.ti. |
| 49 | crossover.ab. |
| 50 | crossover.ti. |
| 51 | cross.ab. |
| 52 | cross.ti. |
| 53 | or/51-52 |
| 54 | over.ab. |
| 55 | over.ti. |
| 56 | or/54-55 |
| 57 | 53 and 56 |
| 58 | exp randomized controlled trial/ |
| 59 | or/28-50,57-58 |
| 60 | 18 and 27 and 59 |
| 61 | limit 60 to human |
| 62 | limit 61 to embase |
| 63 | limit 60 to yr=”2003 - 2013” |
| 64 | limit 61 to yr=”2003 - 2013” |
| 65 | limit 60 to yr=”2013 - 2017” |
| 66 | limit 61 to yr=”2013 - 2017” |
| 67 | limit 60 to yr=”2013 - Current” |
| 68 | limit 61 to yr=”2013 - Current” |

*For lines 67, 68, the search was run in Sep 2019; then again in October 2024

PubMed Search Strategy

| 1 | opioid-related disorders[MeSH Terms] |
| --- | --- |
| 2 | opioid*[Title/Abstract] OR opiat*[Title/Abstract] |
| 3 | opioid*[Text Word] or opiat*[Text Word] |
| 4 | opioid*[All Fields] or opiat*[All Fields] |
| 5 | (opioid*[Title/Abstract] OR opiat*[Title/Abstract]) OR (opioid*[Text Word] OR opiat*[Text Word]) |
| 6 | (opioid*[Title/Abstract] OR opiat*[Title/Abstract]) OR (opioid*[All Fields] OR opiat*[All Fields]) |
| 7 | abus*[Title/Abstract] OR dependen*[Title/Abstract] OR disorder*[Title/Abstract] |
| 8 | abus*[Text Word] OR dependen*[Text Word] OR disorder*[Text Word] |
| 9 | (abus*[Text Word] OR dependen*[Text Word] OR disorder*[Text Word]) OR (abus*[Title/Abstract] OR dependen*[Title/Abstract] OR disorder*[Title/Abstract]) |
| 10 | (abus*[Text Word] OR dependen*[Text Word] OR disorder*[Text Word]) OR (abus*[All Fields] OR dependen*[All Fields] OR disorder*[All Fields]) |
| 11 | (opioid*[All Fields] OR opiat*[All Fields]) AND (abus*[All Fields] OR dependen*[All Fields] OR disorder*[All Fields]) |
| 12 | (opioid*[Title/Abstract] OR opiat*[Title/Abstract]) AND (abus*[Title/Abstract] OR dependen*[Title/Abstract] OR disorder*[Title/Abstract]) |
| 13 | (opioid*[Text Word] OR opiat*[Text Word]) AND (abus*[Text Word] OR dependen*[Text Word] OR disorder*[Text Word]) |
| 14 | (opioid*[All Fields] OR opiat*[All Fields]) AND (abus*[All Fields] OR dependen*[All Fields] OR disorder*[All Fields]) |
| 15 | 12 or 13 |
| 16 | ((opioid*[Title/Abstract] OR opiat*[Title/Abstract]) AND (abus*[Title/Abstract] OR dependen*[Title/Abstract] OR disorder*[Title/Abstract])) OR ((opioid*[All Fields] OR opiat*[All Fields]) AND (abus*[All Fields] OR dependen*[All Fields] OR disorder*[All Fields])) |
| 17 | (opioid-related disorders[MeSH Terms]) OR ((opioid*[All Fields] OR opiat*[All Fields]) AND (abus*[All Fields] OR dependen*[All Fields] OR disorder*[All Fields])) |
| 18 | (opioid-related disorders[MeSH Terms]) OR (((opioid*[Title/Abstract] OR opiat*[Title/Abstract]) AND (abus*[Title/Abstract] OR dependen*[Title/Abstract] OR disorder*[Title/Abstract])) OR ((opioid*[All Fields] OR opiat*[All Fields]) AND (abus*[All Fields] OR dependen*[All Fields] OR disorder*[All Fields]))) |
| 19 | heroin[MESH Terms] |
| 20 | heroin[Title/Abstract] |
| 21 | heroin[All Fields] |
| 22 | (heroin[MESH Terms]) OR (heroin[Title/Abstract]) OR (heroin[All Fields]) |
| 23 | ((heroin[MESH Terms]) OR (heroin[Title/Abstract]) OR (heroin[All Fields])) OR ((opioid*[Title/Abstract] OR opiat*[Title/Abstract]) OR (opioid*[All Fields] OR opiat*[All Fields])) |
| 24 | buprenorphine[MESH Terms] |
| 25 | methadone[MESH Terms] |
| 26 | buprenorphine[Title/Abstract] |
| 27 | buprenorphine[Text Word] |
| 28 | buprenorphine[All Fields] |
| 29 | methadone[Title/Abstract] |
| 30 | methadone[Text Word] |
| 31 | methadone[All Fields] |
| 32 | (buprenorphine[MESH Terms]) OR (methadone[MESH Terms]) OR (buprenorphine[Title/Abstract]) OR (buprenorphine[Text Word]) OR (buprenorphine[All Fields]) OR (methadone[Title/Abstract]) OR (methadone[Text Word]) OR (methadone[All Fields]) |
| 33 | ((opioid-related disorders[MeSH Terms]) OR ((opioid*[All Fields] OR opiat*[All Fields]) AND (abus*[All Fields] OR dependen*[All Fields] OR disorder*[All Fields]))) AND (((heroin[MESH Terms]) OR (heroin[Title/Abstract]) OR (heroin[All Fields])) OR ((opioid*[Title/Abstract] OR opiat*[Title/Abstract]) OR (opioid*[All Fields] OR opiat*[All Fields]))) AND ((buprenorphine[MESH Terms]) OR (methadone[MESH Terms]) OR (buprenorphine[Title/Abstract]) OR (buprenorphine[Text Word]) OR (buprenorphine[All Fields]) OR (methadone[Title/Abstract]) OR (methadone[Text Word]) OR (methadone[All Fields])) |
| 34 | ((opioid-related disorders[MeSH Terms]) OR (((opioid*[Title/Abstract] OR opiat*[Title/Abstract]) AND (abus*[Title/Abstract] OR dependen*[Title/Abstract] OR disorder*[Title/Abstract])) OR ((opioid*[All Fields] OR opiat*[All Fields]) AND (abus*[All Fields] OR dependen*[All Fields] OR disorder*[All Fields])))) AND (((heroin[MESH Terms]) OR (heroin[Title/Abstract]) OR (heroin[All Fields])) OR ((opioid*[Title/Abstract] OR opiat*[Title/Abstract]) OR (opioid*[All Fields] OR opiat*[All Fields]))) AND ((buprenorphine[MESH Terms]) OR (methadone[MESH Terms]) OR (buprenorphine[Title/Abstract]) OR (buprenorphine[Text Word]) OR (buprenorphine[All Fields]) OR (methadone[Title/Abstract]) OR (methadone[Text Word]) OR (methadone[All Fields])) |
| 35 | Limited to 2013-2017 |
| 36 | Limited to 2017-2024 |

PRISMA 2020 Checklist

| **Section and Topic** | **Item #** | **Checklist item** | **Location where item is reported** |
| --- | --- | --- | --- |
| **TITLE** | | |  |
| Title | 1 | Identify the report as a systematic review. | Title page |
| **ABSTRACT** | | |  |
| Abstract | 2 | See the PRISMA 2020 for Abstracts checklist. | Abstract page |
| **INTRODUCTION** | | |  |
| Rationale | 3 | Describe the rationale for the review in the context of existing knowledge. | Page 5, line 79-107 |
| Objectives | 4 | Provide an explicit statement of the objective(s) or question(s) the review addresses. | Page 7, line 110 |
| **METHODS** | | |  |
| Eligibility criteria | 5 | Specify the inclusion and exclusion criteria for the review and how studies were grouped for the syntheses. | Page 7, line 133-148 |
| Information sources | 6 | Specify all databases, registers, websites, organisations, reference lists and other sources searched or consulted to identify studies. Specify the date when each source was last searched or consulted. | Page 6, line 117 |
| Search strategy | 7 | Present the full search strategies for all databases, registers and websites, including any filters and limits used. | Supplementary text |
| Selection process | 8 | Specify the methods used to decide whether a study met the inclusion criteria of the review, including how many reviewers screened each record and each report retrieved, whether they worked independently, and if applicable, details of automation tools used in the process. | Page 8, line 149 |
| Data collection process | 9 | Specify the methods used to collect data from reports, including how many reviewers collected data from each report, whether they worked independently, any processes for obtaining or confirming data from study investigators, and if applicable, details of automation tools used in the process. | Page 8, line 149 |
| Data items | 10a | List and define all outcomes for which data were sought. Specify whether all results that were compatible with each outcome domain in each study were sought (e.g. for all measures, time points, analyses), and if not, the methods used to decide which results to collect. | Page 9, line 166-171 |
|  | 10b | List and define all other variables for which data were sought (e.g. participant and intervention characteristics, funding sources). Describe any assumptions made about any missing or unclear information. | Page 9, line 166-171 |
| Study risk of bias assessment | 11 | Specify the methods used to assess risk of bias in the included studies, including details of the tool(s) used, how many reviewers assessed each study and whether they worked independently, and if applicable, details of automation tools used in the process. | Page 8, 157-164 |
| Effect measures | 12 | Specify for each outcome the effect measure(s) (e.g. risk ratio, mean difference) used in the synthesis or presentation of results. | Page 9, line 184-188 |
| Synthesis methods | 13a | Describe the processes used to decide which studies were eligible for each synthesis (e.g. tabulating the study intervention characteristics and comparing against the planned groups for each synthesis (item #5)). | Page 9, line 177-179 |
|  | 13b | Describe any methods required to prepare the data for presentation or synthesis, such as handling of missing summary statistics, or data conversions. | Page 9, line 177-179 |
|  | 13c | Describe any methods used to tabulate or visually display results of individual studies and syntheses. | Page 9, line 173-176 |
|  | 13d | Describe any methods used to synthesize results and provide a rationale for the choice(s). If meta-analysis was performed, describe the model(s), method(s) to identify the presence and extent of statistical heterogeneity, and software package(s) used. | Page 9, line 173-183 |
|  | 13e | Describe any methods used to explore possible causes of heterogeneity among study results (e.g. subgroup analysis, meta-regression). | Page 10, 196-204 |
|  | 13f | Describe any sensitivity analyses conducted to assess robustness of the synthesized results. | Page 10, 196-204 |
| Reporting bias assessment | 14 | Describe any methods used to assess risk of bias due to missing results in a synthesis (arising from reporting biases). | Funnel plots |
| Certainty assessment | 15 | Describe any methods used to assess certainty (or confidence) in the body of evidence for an outcome. | None |
| **RESULTS** | | |  |
| Study selection | 16a | Describe the results of the search and selection process, from the number of records identified in the search to the number of studies included in the review, ideally using a flow diagram. | Page 11, line 207-220 |
|  | 16b | Cite studies that might appear to meet the inclusion criteria, but which were excluded, and explain why they were excluded. | Figure 1 |
| Study characteristics | 17 | Cite each included study and present its characteristics. | Table 1 |
| Risk of bias in studies | 18 | Present assessments of risk of bias for each included study. | Supplementary table |
| Results of individual studies | 19 | For all outcomes, present, for each study: (a) summary statistics for each group (where appropriate) and (b) an effect estimate and its precision (e.g. confidence/credible interval), ideally using structured tables or plots. | NA |
| Results of syntheses | 20a | For each synthesis, briefly summarise the characteristics and risk of bias among contributing studies. | Page 13, line 229-237 |
|  | 20b | Present results of all statistical syntheses conducted. If meta-analysis was done, present for each the summary estimate and its precision (e.g. confidence/credible interval) and measures of statistical heterogeneity. If comparing groups, describe the direction of the effect. | Page 15 onwards |
|  | 20c | Present results of all investigations of possible causes of heterogeneity among study results. | Page 15 onwards |
|  | 20d | Present results of all sensitivity analyses conducted to assess the robustness of the synthesized results. | Page 15 onwards |
| Reporting biases | 21 | Present assessments of risk of bias due to missing results (arising from reporting biases) for each synthesis assessed. | None |
| Certainty of evidence | 22 | Present assessments of certainty (or confidence) in the body of evidence for each outcome assessed. | None |
| **DISCUSSION** | | |  |
| Discussion | 23a | Provide a general interpretation of the results in the context of other evidence. | Page 20, line 387-408 |
|  | 23b | Discuss any limitations of the evidence included in the review. | Page 22, line 437-444 |
|  | 23c | Discuss any limitations of the review processes used. | Page 22, line 433-436 |
|  | 23d | Discuss implications of the results for practice, policy, and future research. | Page 21, 23, line 409-423, 445-455. |
| **OTHER INFORMATION** | | |  |
| Registration and protocol | 24a | Provide registration information for the review, including register name and registration number, or state that the review was not registered. | Page 6, line 119 |
|  | 24b | Indicate where the review protocol can be accessed, or state that a protocol was not prepared. | Page 6, line 119 |
|  | 24c | Describe and explain any amendments to information provided at registration or in the protocol. | NA |
| Support | 25 | Describe sources of financial or non-financial support for the review, and the role of the funders or sponsors in the review. | In submission portal |
| Competing interests | 26 | Declare any competing interests of review authors. | In submission portal |
| Availability of data, code and other materials | 27 | Report which of the following are publicly available and where they can be found: template data collection forms; data extracted from included studies; data used for all analyses; analytic code; any other materials used in the review. | In submission portal |

*From:*  Page MJ, McKenzie JE, Bossuyt PM, Boutron I, Hoffmann TC, Mulrow CD, et al. The PRISMA 2020 statement: an updated guideline for reporting systematic reviews. BMJ 2021;372:n71. doi: 10.1136/bmj.n71

# S2 Text. Additional results

We considered a third network where personalized treatment regimens were treated as separate nodes from fixed treatment regimens (e.g., fixed dose methadone, individually titrated methadone). However, there are two issues with defining nodes by personalized regimens. Firstly, as we noted in the results of our literature review, earlier trials tended to examine fixed regimens whereas later trials tended to examine of personalized regimens. This may introduce confounding by way of period effects (e.g., standard of care beyond pharmaceutical treatment that may differ between earlier and more recent trials). Second, recent treatment guidelines of OUD with conventional OAT generally favour individually titrating dosages (see examples from the WHO International Standards for the Treatment of Drug Use Disorders,[62] American Society for Addiction Medicine,[59] national guidance CIHR Canadian Research Initiative in Substance Misuse,[63] and the UK Department of Health & Social Care guidelines[64]). Nonetheless, analyses based on treatment nodes separated by dosing variability are contained within the Supplementary Files (**Tables** **9-11**).
